# Nuciferol C, a new sesquineolignan dimer from *Cocos nucifera* L.: bioactivity and theoretical investigation†

**DOI:** 10.1039/d4ra02940b

**Published:** 2024-08-16

**Authors:** Marwa Elsbaey, Yasuhiro Igarashi, Radwan Alnajjar, Khaled M. Darwish, Tomofumi Miyamoto

**Affiliations:** a Pharmacognosy Department, Faculty of Pharmacy, Mansoura University Mansoura 35516 Egypt marwaelsebay1611@mans.edu.eg; b Biotechnology Research Center and Department of Biotechnology, Toyama Prefectural University 5180 Kurokawa, Imizu Toyama 939-0398 Japan; c CADD Unit, Faculty of Pharmacy, Libyan International Medical University Benghazi 16063 Libya; d Department of Medicinal Chemistry, Faculty of Pharmacy, Suez Canal University Ismailia 41522 Egypt; e School of Pharmaceutical Sciences, Kyushu University 3-1-1 Maidashi, Higashi-ku Fukuoka 812-8582 Japan

## Abstract

Nuciferol C (NC), an undescribed dimer of nuciferol B (NB), was isolated from the endocarp of *Cocos nucifera* L. The planar structure of NC was determined using 1D- and 2D-NMR spectroscopy as well as high resolution MS spectrometry. The absolute configuration was concluded based on analysis of NOESY spectra. NC showed cytotoxic activity against colon cancer cells (CaCo-2) with an IC_50_ value of 76 μM, and significantly decreased the expression of human epidermal growth factor receptor (EGFR) and tumor necrosis factor alpha (TNF-α) in CaCo-2 as compared with untreated cells by 39% and 33%, respectively (*p* < 0.05). In addition, NC exhibited anti-herpes simplex virus (HSV-I) activity with an IC_50_ value of 23 μM. *In silico* study of NC was implemented at three levels: density functional theory (DFT) was used to study its electronic properties, molecular mechanics was used to estimate the docking results, and finally, molecular dynamic simulation was used to study the behavior and stability of NC inside the active site of the target protein of HSV-1.

## Abstract 2

Nuciferol C (NC), an undescribed dimer of nuciferol B (NB), was isolated from the endocarp of *Cocos nucifera* L.

## Graphical abstract

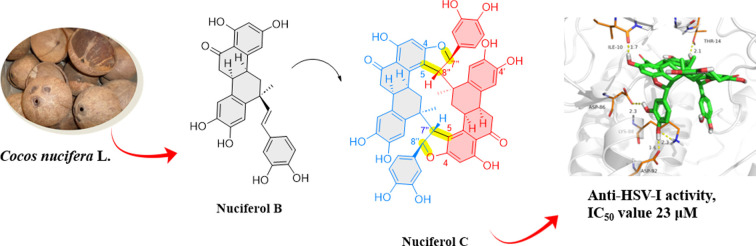

## 1. Introduction

*Cocos nucifera* L. var. *typica* (Arecaceae), commonly known as coconut palm, is an important economic crop in the food and cosmetics industry.^1^ In 2020, global coconut production was estimated at 11.7 million tons.^2^ A coconut consists of the outer epicarp (husk), the mesocarp (husk fibers), the inner endocarp, and the edible fleshy endosperm (coconut meat).^1^ Almost every part of the coconut is valorized: the edible part for its nutritional value, coconut oil is for its cosmetic products,^2^ the husks as a resource for biofuel,^3^ and the fibers for their textile applications.^4^ However, in some countries, the inefficient consumption of the non-edible parts accumulates about 62–65% of coconut production as tons of waste, releasing considerable amounts of airborne pollutants.^5^ In this context, recycling coconut waste as a sustainable source for bioactive metabolites is of environmental and economic merit.

In addition, numerous bioactivities have been attributed to the different parts of coconut waste, including cytotoxic, antineoplastic, antiviral, antimicrobial, antibiofilm, antimalarial, analgesic, anti-inflammatory, vasorelaxant, antihypertensive and thrombolytic activities.^1,6,7^ Despite the multitude of the reported bioactivities, few studies have addressed the phytoconstituents of coconut parts. For instance, catechins and flavonoids were reported from the green husk and the husk fibers.^1,8^ For the endocarp, no phytochemical investigations have been reported up to 2017, when we started our phytochemical investigations. We have previously isolated phenylpropanoids, lignans, stilbenes, stilbene dimers, flavonoids and phenolic acids from the endocarp of *C. nucifera*.^6,9,10^ One of our studies reported the novel cyclosesquineolignan epimers, nuciferols A (NA) and B (NB).^10^ The skeleton of nuciferols consisted of three phenyl propane (C6–C3) units connected together *via* 6.9′, 9.6′, 7′.8′′-linkage and showed an unusual migration of C-9′′ from one unit to the other precisely from C-8′′ to C-7′.^10^ Herein, we report the isolation, structure elucidation, bioactivity and theoretical investigation of another dimeric cyclosesquineolignan from *C. nucifera*.

## 2. Results and discussion

### 2.1. Structure elucidation

**Fig. 1:**
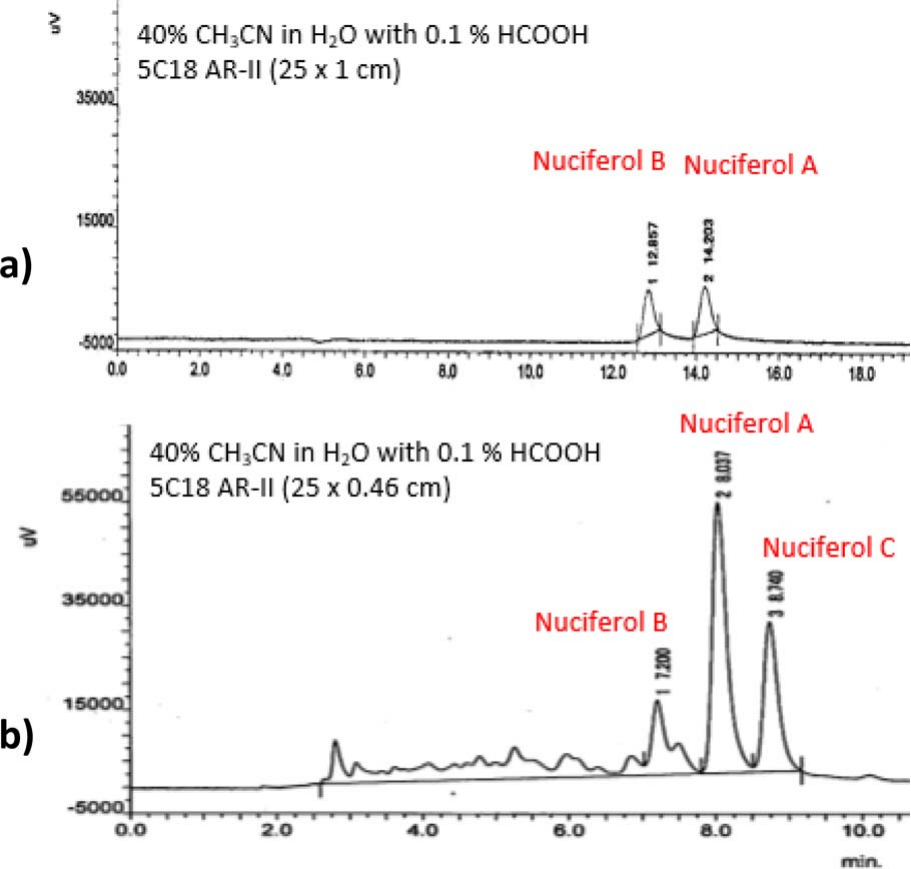
HPLC chromatogram of nuciferols. (a) Former HPLC analysis. (b) HPLC analysis after storage.

The ethyl acetate (EtOAc) extract of *C. nucifera* endocarp was fractionated on a silica gel column with elution by *n*-hexane-EtOAC mixtures. The eluate with *n*-hexane-EtOAC (3/7) was further subjected to Sephadex LH-20 and silica gel with elution by CHCl_2_–MeOH mixtures to afford an isomeric mixture of nuciferols, of which NA and NB were obtained by reversed phase HPLC (Fig. 1a).^10^ Remnants of this mixture were stored in a sealed glass bottle at room temperature for approximately six months. Reinvestigation of these remnants by high performance liquid chromatography with diode-array detection (HPLC-DAD) analysis revealed the presence of three peaks (Fig. 1b) instead of two, like our former HPLC analysis (Fig. 1a).^10^

**Fig. 2:**
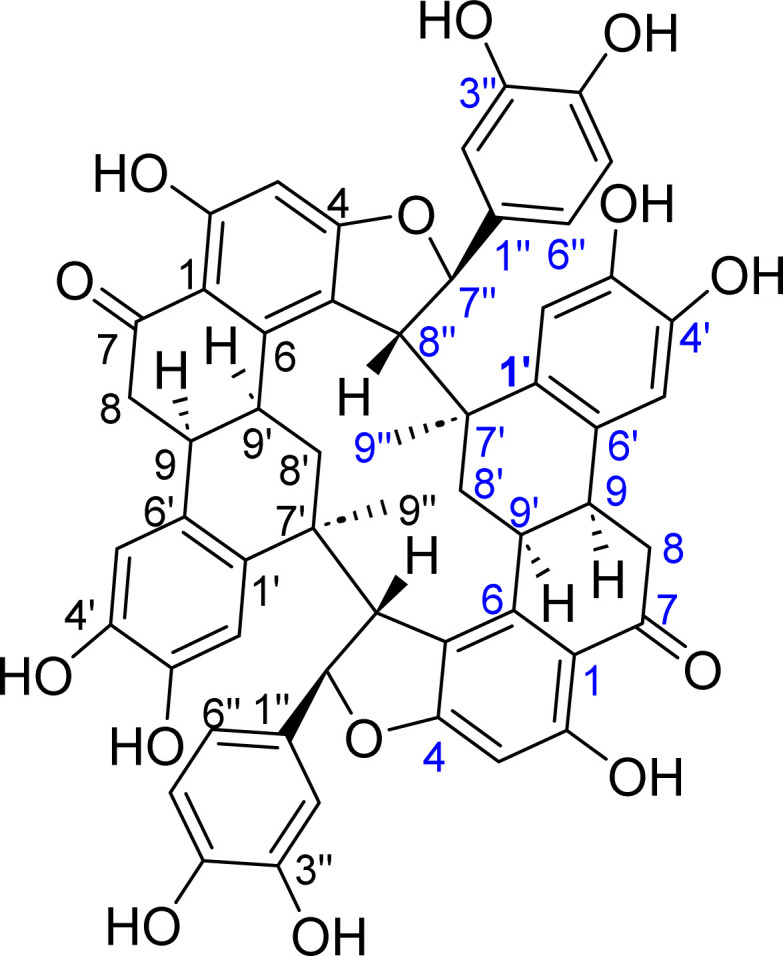
Structure of nuciferol C (NC) isolated from *Cocos nucifera* L.

Hence, the constituents of the mixture were separated by reversed phase HPLC using 40% CH_3_CN in H_2_O containing 0.1% formic acid (Fig. 1b) and each peak was subjected to NMR analysis. The first two peaks (0.1 mg, *t*_R_ 7.2 min) and (0.8 mg, *t*_R_ 8.0 min) were identified as NB and NA, respectively, by comparison of their ^1^H-NMR data (Fig. S12, S13 and Table S1†) to nuciferols,^10^ meanwhile the third peak (0.9 mg, *t*_R_ 8.7 min) was deduced as a dimer of NB and hence designated nuciferol C (NC) (Fig. 2). Herein, the detailed structure elucidation of NC.

**Fig. 3:**
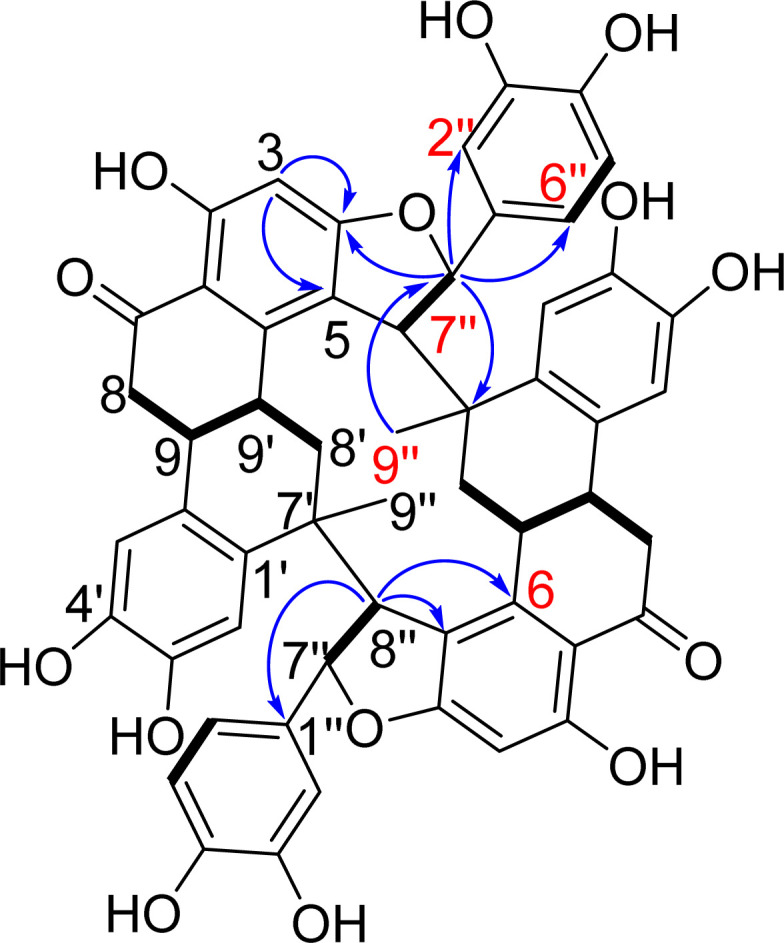
Key COSY and HMBC correlations for NC.

Nuciferol C (NC) was purified as a reddish-brown amorphous solid, [*α*]^24^_D_ + 6 (*c* 0.1, MeOH). The molecular formula was deduced as C_54_H_44_O_14_ based on analysis of NMR and MS data. HRESIMS showed a deprotonated molecular ion peak at *m*/*z* 915.2656 [M−H]^−^ (calcd for C_54_H_43_O_14_, 915.2658), indicating 32 degrees of unsaturation. ^1^H-NMR and HSQC (Table 1) displayed a tertiary methyl group (*δ*_H_ 1.24, s), two methylenes (*δ*_H_ 2.00/2.18 and 3.06), four methines (*δ*_H_ 3.31, 3.38, 3.54 and 5.34), and six aromatic protons (*δ*_H_ 5.94, 6.56, 6.74, 6.86, 6.98 and 7.08), assigned to three aromatic ring systems as revealed by COSY and HMBC (Table 1 and Fig. 3). ^13^C-NMR spectrum exhibited twenty-seven carbons assignable to eighteen aromatic and nine aliphatic carbons, indicating the presence of three C6–C3 units of a sesquilignan skeleton. NMR data inferred structural similarity to nuciferols^10^ except for the replacement of the *trans*-olefinic protons (H-7′′ and H-8′′) by two vicinal methines *δ*_H_ 5.34/*δ*_c_ 93.5 and *δ*_H_ 3.54/*δ*_c_ 59.9 and the disappearance of the *meta*-coupled proton (H-5) (Table S1†). The downfield shift of the proton signal at *δ*_H_ 5.34/*δ*_C_ 93.5 indicated it is attached to oxygen. The key HMBC correlation of H-8′′ to C-5 and C-6 and of H-7′′ to C-4 suggested that C-7′′, C-8′′, C-4 and C-5 are incorporated in a furan ring. The previous data indicated 16 degrees of unsaturation out of 32, suggesting the presence of a symmetric dimer.

**Table 1:** ^13^C (150 MHz) and ^1^H (600 MHz) NMR data for NC in CD_3_OD

H/C	*δ*_C_, mult.	*δ*_H_ (*J* in Hz)	HMBC
1, 1	112.9, C	—	
2, 2	168.4, C	—	
3, 3	97.5, CH	5.94, s	1, 2, 4, 5
4, 4	165.3, C	—	
5, 5	124.4, C	—	
6, 6	144.8, C	—	
7, 7	200.8, C	—	
8, 8	43.6, CH_2_	3.06, m	6′, 7, 9′
9, 9	42.7, CH	3.38, m	
1′, 1′	132.63, C	—	
2′, 2′	117.9, CH	6.74, s	4′, 6′, 7′
3′, 3′	144.0, C	—	
4′, 4′	144.9, C	—	
5′, 5′	114.7, CH	6.56, s	1′, 3′, 9
6′, 6′	131.7, C	—	
7′, 7′	40.1, C	—	
8′α, 8′α	43.2, CH_2_	2.00, d (13.2)	6, 9, 7′, 9′, 8′′, 9′′
8′β, 8′β	—	2.18, dd (13.2, 5.4)	9, 1′, 8′′
9′, 9′	36.0, CH	3.31^a^	7′
1′′, 1′′	132.64, C	—	
2′′, 2′′	115.5, CH	7.08, d (2.0)	4′′, 6′′, 7′′
3′′, 3′′	146.8, C	—	
4′′, 4′′	147.3, C	—	
5′′, 5′′	116.3, CH	6.86, d (8.1)	1′′, 3′′, 6′′
6′′, 6′′	120.6, CH	6.98, dd (8.1, 2.1)	2′′, 4′′, 7′′
7′′, 7′′	93.5, CH	5.34, d (9.9)	7′, 2′′, 6′′, 8′′
8′′, 8′′	59.9, CH	3.54, d (9.9)	5, 6, 8′, 9′, 1′′, 7′′
9′′, 9′′	26.7, CH_3_	1.24, s	1′, 7′, 8′, 8′′

a Overlapped with solvent signal, determined through HSQC.

Since NC is a symmetric dimer, it was proposed as a dimer of either NA or NB. However as shown in the HPLC chromatogram (Fig. 1), NA remained in the mixture, meanwhile NB was merely present, inferring that NC may be derived from NB. As shown in Fig. S14,† dimerization could have proceeded *via* intermolecular coupling of the alkene bond and the phenolic part allowing the formation of a benzofuran derivative, similar to dimerization of stilbenes.^11^ Accordingly, it is possible that dimerization was induced by light or oxidation. Kosović *et al.* reported light induced dimerization of *trans*-resveratrol in grapevine extracts.^12^ Similarly, Langcake and Pryce described the *in vitro* production of oligomeric stilbenes in grapevine upon UV irradiation.^11^ In addition, biomimetic synthesis of stilbene dimers has been achieved by several oxidizing agents.^13^

**Fig. 4:**
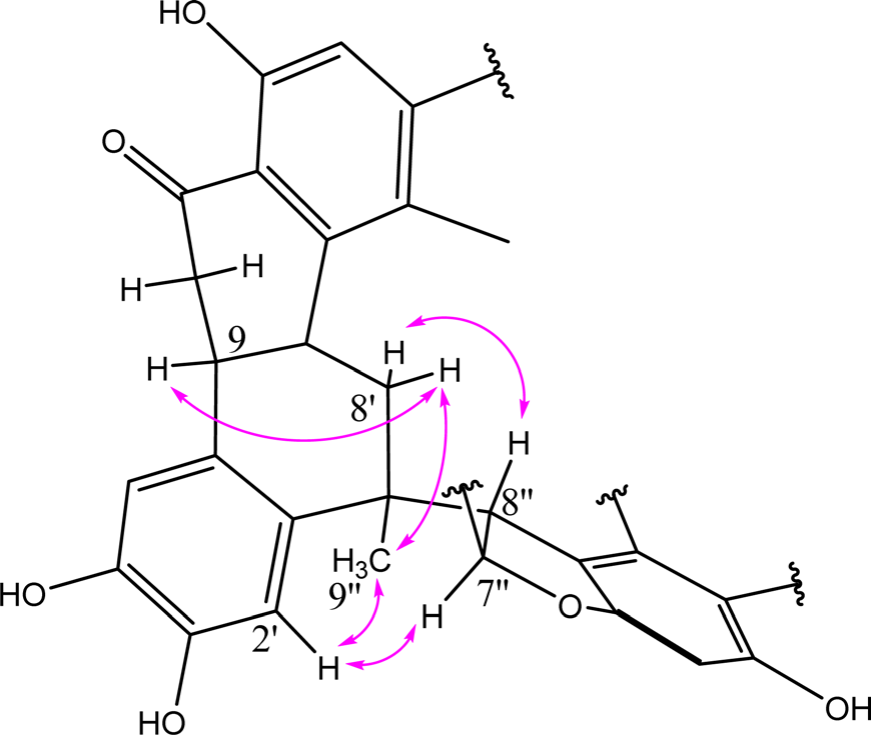
Key NOESY correlations for NC.

The former data established the planar structure of NC. The relative configuration was deduced from NOESY correlations (Fig. 4). Previously, we have proved the absolute configurations of nuciferols,^10^ including the *S*-configuration of H-9 and H-9′. The correlation from H-9α to H-8′α (*δ*_H_ 2.00) indicated its α-orientation and, hence the β-orientation of H-8′β (*δ*_H_ 2.18). The β-orientation of H-8′′ was concluded from its NOESY correlation to H-8′β. The *trans*-configuration of the alkene bond in nuciferols suggested that H-7′′ and H-8′′ in NC are on opposite sides of the ring,^14^ also concluded from the value of the coupling constant (*J*_7′′8′′_ = 9.9).^15^ Accordingly, H-7′′ was concluded to be α-directed. The NOESY correlations from H-7′′α to H-2′, from H-2′ to CH_3_-9′′ and from CH_3_-9′′ to H-8′α proved their α-direction and suggested NC as an NB dimer. Accordingly, this divulged the absolute configuration of NC to be 9*S*, 7′*S*, 9′*S*, 7′′*R*, 8′′*R*.

### 2.2. Cytotoxic activity

Plant-derived lignans exhibit various pharmacological activities, of which the anticancer activity is the most remarkable. The clinically important anticancer drugs etoposide and teniposide are semisynthetic derivatives of the lignan podophyllotoxin.^16^ In addition, studies suggested that dietary intake of lignans is linked with a reduced risk of colon cancer and gastric adenocarcinoma.^16^ Hence, NC was investigated against CaCo-2 colon cancer cells. NC displayed cytotoxic effect with an IC_50_ value of 27 μM. In addition, it was further investigated for its anticancer properties. Caco-2 cells, incubated with the cytotoxic concentration of NC for 24 h, displayed a significantly reduced level of EGFR and TNF-α as compared with untreated cells, by 39 and 33%, respectively (*p* < 0.05). Targeting EGFR and TNF-α is among the most successful strategies to be exploited in cancer therapy. EGFR is a key driving molecule for cell division, apoptosis, cell differentiation, invasion and migration. It is overexpressed in many tumors including colon and colorectal cancer.^17^ Meanwhile, TNF-α is an inflammatory cytokine that promotes cancer cell migration, invasion and colon cancer metastasis.^18^ In this context, NC may have a preventive effect against colon cancer migration and metastasis. The findings indicate that NC can be recommended for further investigations for its anticancer effects.

### 2.3. Antiviral activity

Podophyllotoxin and bicyclol are well known lignans for their powerful antiviral action in treatment of papillomavirus and chronic hepatitis B, respectively.^19^ Accordingly, this prompted us to investigate the antiviral potential of NC. The half-maximal cytotoxic concentration (CC_50_) and the maximum nontoxic concentration (MNTC) of NC were determined on Vero cells using MTT assay (Table S2†). Serial dilutions of the MNTC of NC were tested for the antiviral activity against HSV-I-infected Vero cells, where NC showed anti-HSV-I activity with an IC_50_ value of 23 μM. NC inhibited HSV-1 replication without apparent cytotoxicity on cells showing a selectivity index (SI) value of 3.2, and indicating its antiviral potential. These findings are in agreement with data reported for other plant-derived lignans, where yatein is also reported to inhibit HSV-1 replication and DNA synthesis.^19^ In order to postulate the antiviral mechanism of NC, *in silico* studies were conducted against the active site of the target protein of HSV-1.

### 2.4. *In silico* studies

**Fig. 5:**
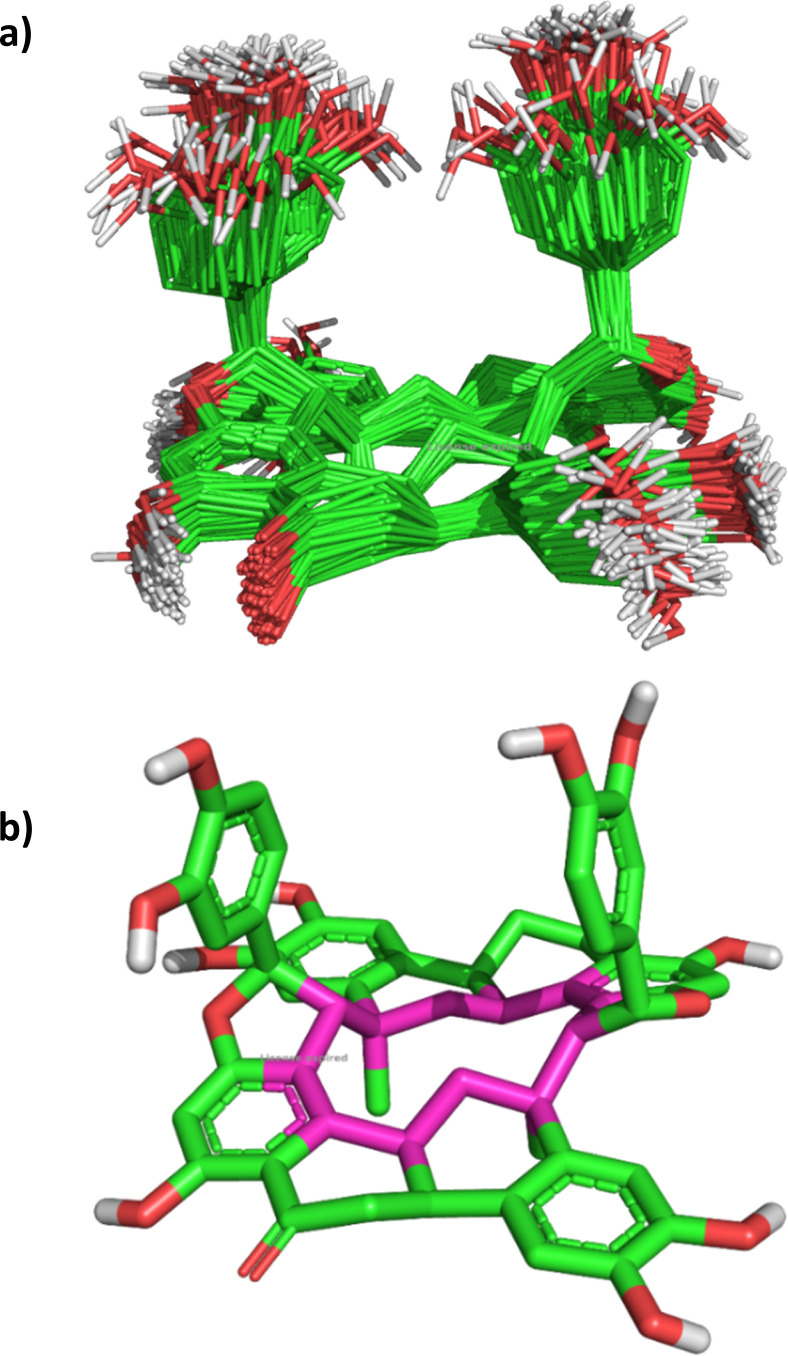
The conformation of NC during the molecular dynamic simulation: (a) clustering every 10 ns; (b) snapshot at 100 ns.

The 3D structure of NC was first subjected to molecular dynamics simulation for 100 ns to establish the conformation of the cyclododecane. The obtained results were clustered into one file with 10 ns step each, and it was found that the cyclododecane prefers a conformation where the two phenyl groups are perpendicular to the cyclododecane, Fig. 5a. The furan ring restricts the conformation of the cyclododecane for adopting the well-known square conformation, Fig. 5b.^20–22^

**Fig. 6:**
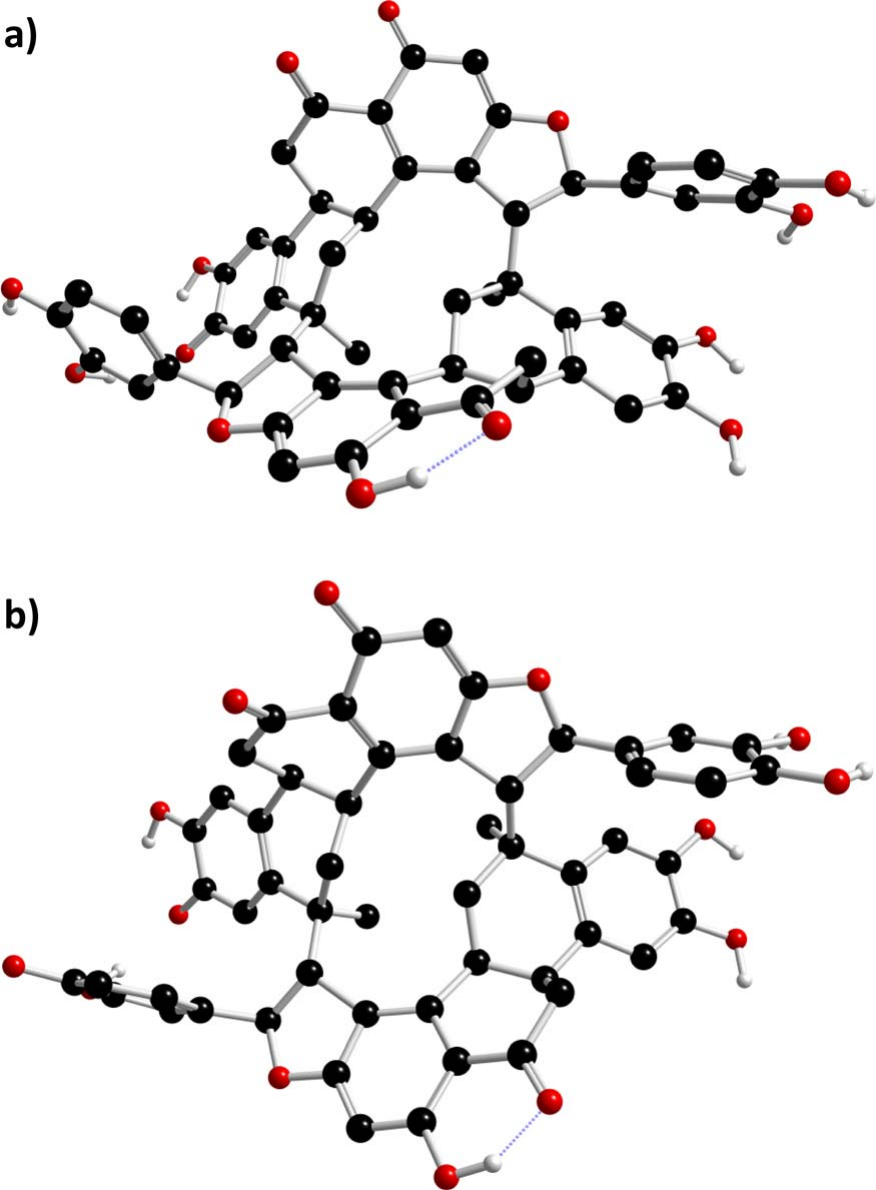
The structure of NC obtained at B3LYP/6-31+G*/Gas level; (a) side view; (b) top view.

Once the conformation of NC was obtained, DFT^23^ calculations were implemented to get a more accurate structure. The well-known B3LYP/6-31+G* level^24,25^ in the gas phase was used, and it was found that the cyclododecane had a different conformation than that was obtained from the MD simulation. The obtained structure is depicted in Fig. 6.

**Fig. 7:**
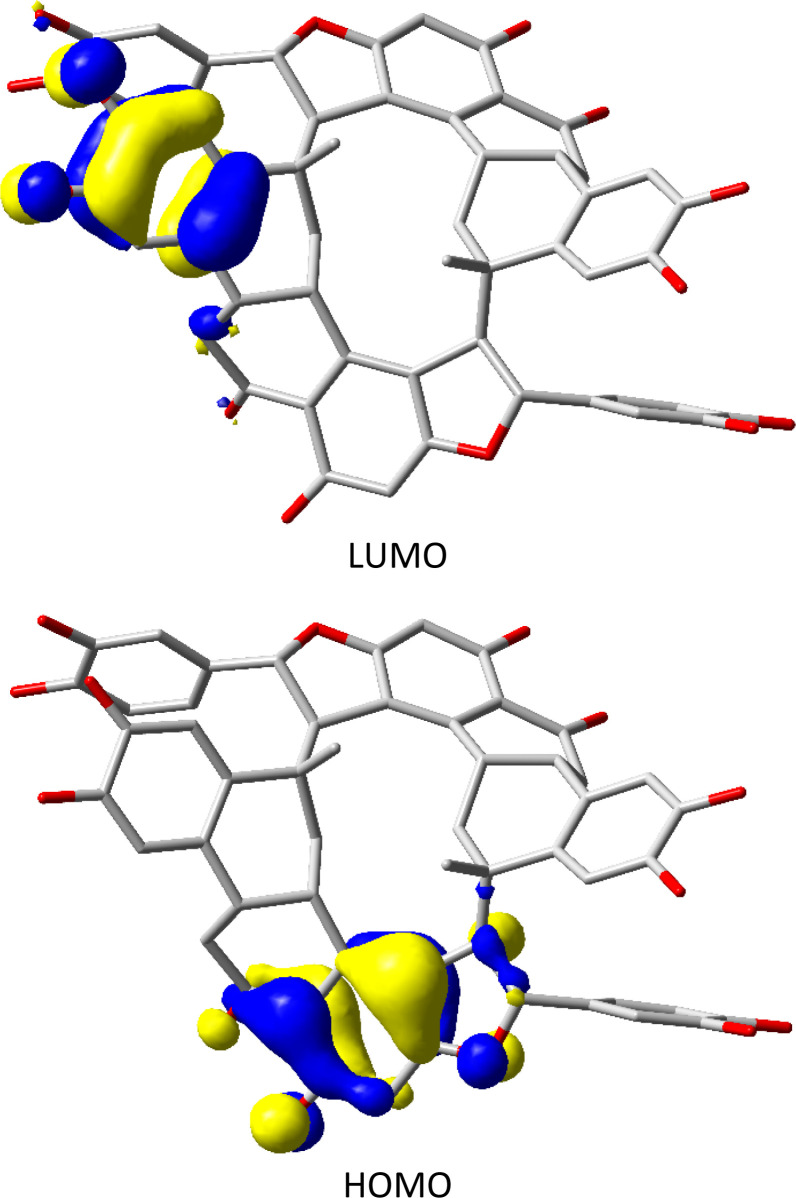
Frontier molecular orbitals of NC. Energy gap (*E*_g_): 0.2214 eV.

The frontier molecular orbitals of NC were also investigated, and it was found that most of the electronic density of the highest occupied molecular orbital (HOMO) is concentered around the benzo group that is next to the furan ring, while the lowest unoccupied molecular orbital is clouded on the other side benzo group, Fig. 7.

**Fig. 8:**
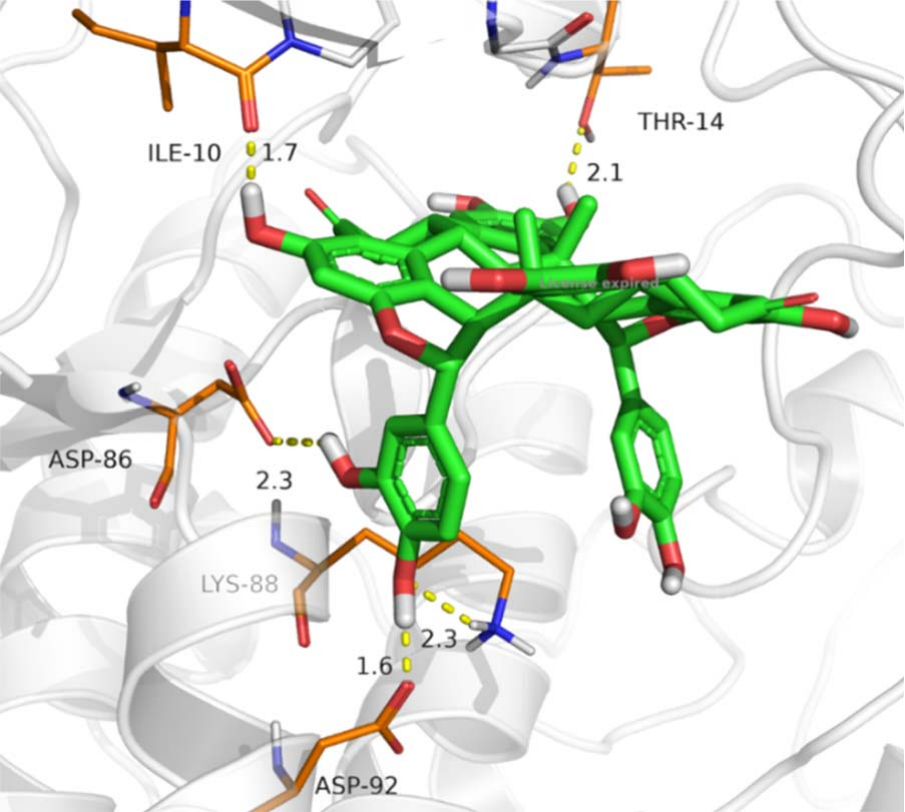
H-bonds interaction of NC with the active site of CDK 2.

To get a deep understanding of the mechanism of action of NC against HSV-I, a docking study was conducted against the active site of both herpes simplex type-1 thymidine kinase and cyclin-dependent kinase 2 (CDK 2). The docking against thymidine kinase was unsuccessful; NC was quite large and rigid; hence, it could not fit inside the tight active site of thymidine kinase, and the docking results were unfavored (positive scores). This finding suggested that if NC is active against herpes simplex type-1 thymidine kinase, it may have a different mechanism of action than acyclovir, and it may involve allosteric inhibition. Since our *in vitro* study of NC showed inhibition activity against Vero cells, CDK 2 was suggestkinase 2 was suggested as a target, as it plays an essential role suggested as a target, as it plays an essential role in the replication of the HSV. The docking result of NC into the active site of the CDK 2 is presented in Table 2. NC showed a docking result of −7.44 kcal mol^−1^ compared to −8.52 kcal mol^−1^ of roscovitine, and was able to from up to five H-bonds toward Thr14 (2.14 Å), Ile10 (1.66 Å), Asp86 (2.34 Å), Lys88 (2.32 Å), and Asp92 (1.55 Å), these interactions are depicted in Fig. 8.

**Table 2:** Docking score and interaction type of NC inside the active side of CDK 2

	Docking score	Residue	Distance
NC	−7.45	Thr14/H-bond	2.14
		Ile10/H-bond	1.66
		Asp86/H-bond	2.34
		Lys88/H-bond	2.32
		Asp92/H-bond	1.55
Roscovitine	−8.52	—	—

**Fig. 9:**
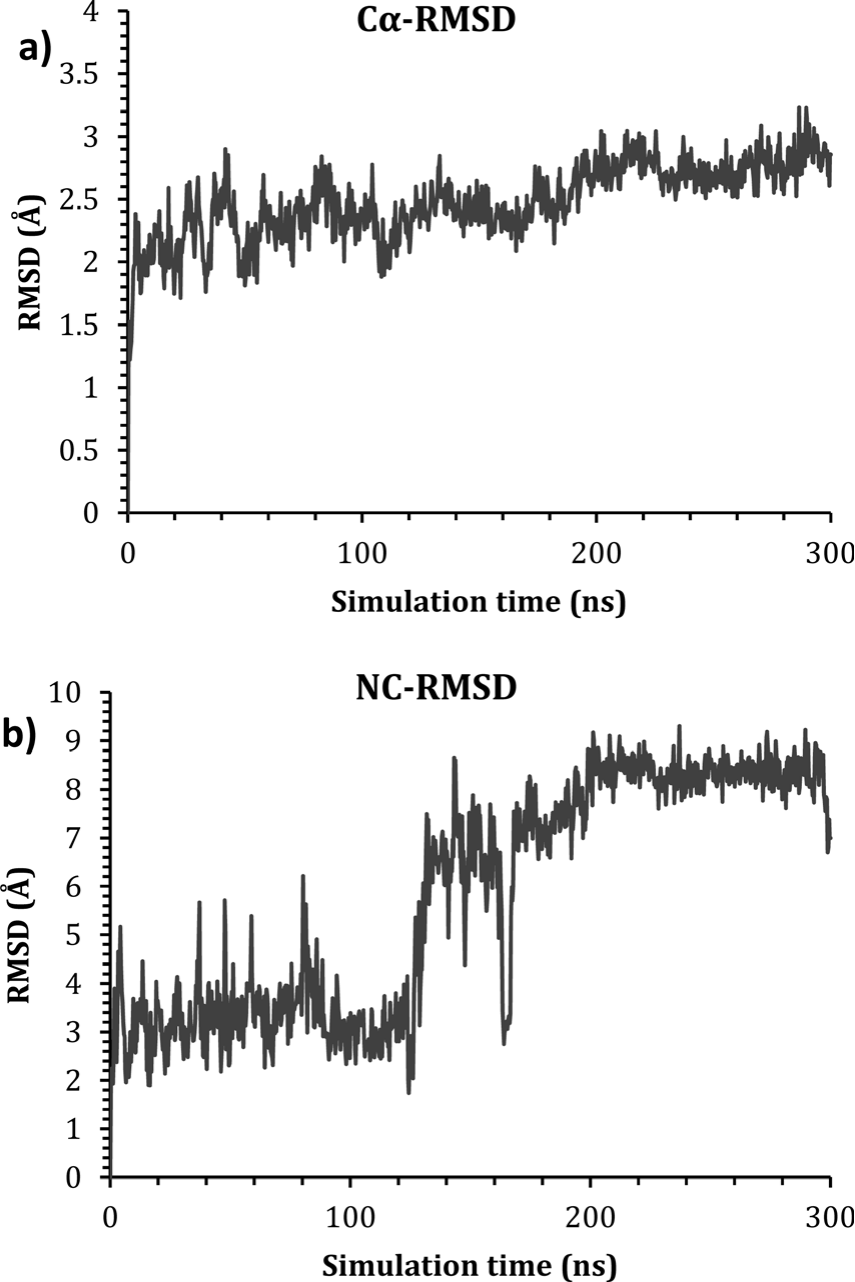
The RMSD of (a) the C_α_ of the ligand–protein complex; (b) ligand with respect to the active site.

Docking results are considered unreliable due to the fact that protein atoms lack movement. Hence, a more accurate technique is desirable.^26,27^ In this regard, molecular dynamics simulation was implemented for 300 ns to study the behavior of NC with the active site of CDK 2. The stability of NC inside the active site was measured by monitoring the RMSD of the protein backbone with respect to its initial position as a function of simulation time, Fig. 9a. In addition, the RMSD of the ligand with respect to its initial position inside the active site was also measured as a function of time, Fig. 9b.

**Fig. 10:**
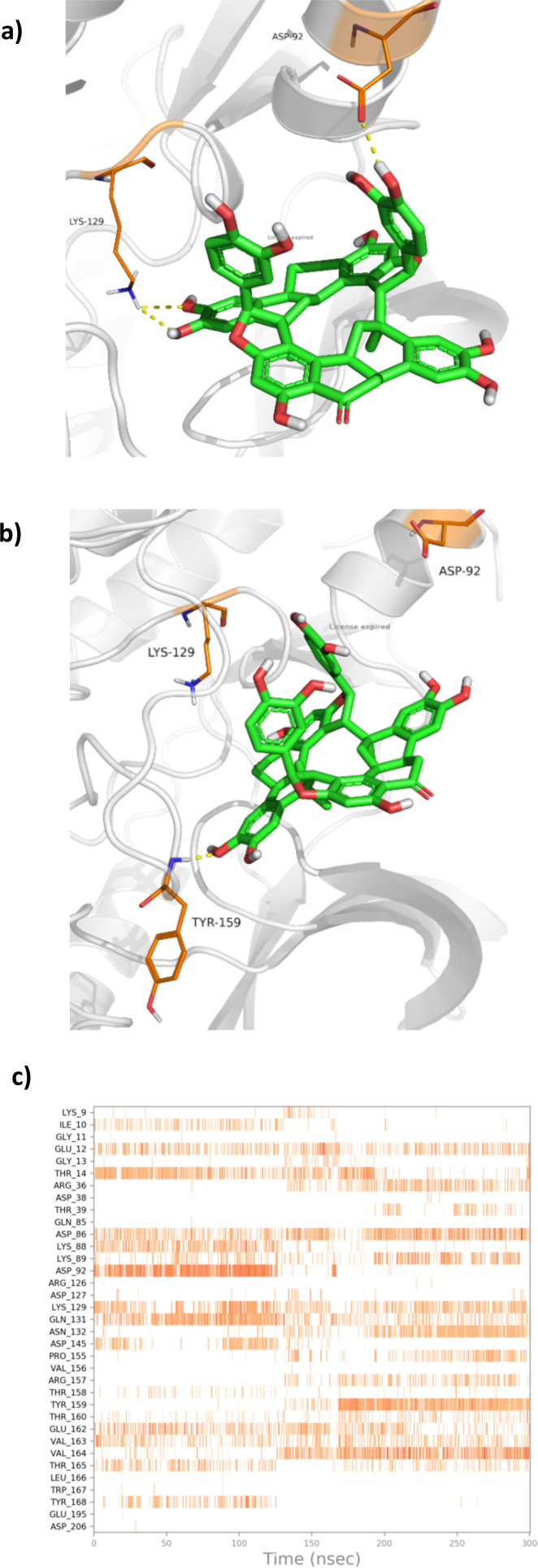
The interaction between NC and CDK 2 at (a) 0 ns, (b) 300 ns, (c) the heat map of the interaction as a function of time.

As is seen in Fig. 9a, the protein C_α_ atoms were stable with an RMSD of 2.50–3.00 Å, which is acceptable for such a protein.^28,29^ The RMSD of the ligand, on the other side, showed stability from the beginning of the simulation till around 120 ns at 3.50 Å, before it fluctuated and moved by around 5.00 Å to stabilize at around 9.00 Å till the end of the simulation. This fluctuation is due to the fact that NC lost its interaction with Asp92 at around 120 ns and formed a new interaction with Tyr159, as can be seen in Fig. 10a–c.

## 3. Experimental

### 3.1. General experimental procedure

Optical rotation measurement was done on a JASCO P-1030 polarimeter at 24 °C. IR and UV spectra were recorded on a Bruker ALPHA II FTIR Spectrometer and a Shimadzu U-1601PC spectrophotometer, respectively. NMR spectra were obtained on a Varian INOVA spectrometer (600 MHz). HRESITOFMS was measured with a Bruker microTOF mass spectrometer. Chromatographic separations were carried out using Silica gel G 60-230 (Merck, Germany) and Sephadex LH-20 (Sigma-Aldrich, Missouri, USA). HPLC was performed using Cosmosil 5C18AR-II, 25 × 1 cm i.d and 25 × 0.46 cm i.d with a Jasco PU2089 gradient pump and PU2075 UV/VIS detector. Thin-layer chromatography was carried out using Merck precoated silica gel F254 plates and vanillin–sulfuric acid spray reagent.

### 3.2. Plant material

The supplier of *Cocos nucifera* L. var. *typica* (Tall) and the details of extraction, fractionation and isolation were previously described.^6,9,10^ The powdered endocarp (8.5 kg) was extracted with MeOH (7 × 10 L). The combined extracts were evaporated *in vacuo* to afford ≈420 g, which were dissolved in 50% aqueous MeOH and partitioned with *n*-hexane, CH_2_Cl_2_, EtOAC and *n*-BuOH. The EtOAC extract (≈38 g) was chromatographed on a silica gel column with elution mixtures of [0–100%] EtOAC in *n*-hexane then [0–20%] MeOH in EtOAC to afford four fractions. Fraction 2 (1.3 g, eluted with 70% EtOAC in *n*-hexane) was gel-filtered on a Sephadex LH-20 to yield subfraction-2 (23.6 mg, eluted with 12.5% MeOH in CH_2_Cl_2_). It was chromatographed on a silica gel column eluted with 3% MeOH in CH_2_Cl_2_ to yield 12.8 mg of the isomeric nuciferols, the former of which were finally purified by reversed phase HPLC (Cosmosil 5C18 AR-II, 25 × 1 cm) with 40% CH_3_CN in H_2_O containing 0.1% formic acid to give NB (4.4 mg, *t*_R_ 12.8 min) and NA (5.6 mg, *t*_R_ 14.2 min).^10^ The remnants of this fraction (2.5 mg) were stored in a sealed glass bottle at room temperature for approximately a six-month period. Final purification of these remnants was performed by reversed phase HPLC (Cosmosil 5C18 AR-II, 25 × 0.46 cm) with 40% CH_3_CN in H_2_O containing 0.1% formic acid to give NB (0.1 mg, *t*_R_ 7.2 min), NA (0.8 mg, *t*_R_ 8.0 min) and NC (0.9 mg, *t*_R_ 8.7 min).

Nuciferol C (NC): reddish-brown amorphous; [*α*]^24^_D_ + 6 (*c* 0. 1, MeOH); IR (ATR) *ν*_max_ 3391, 2920, 2885, 1627, 1422, 1390 cm^−1^; UV (MeOH) *λ*_max_ (log *ε*) 220 (4.52), 281 (4.18) nm; HRESIMS *m*/*z* 915.2656 [M−H]^−^ [calcd for C_54_H_43_O_14_, 915.2658].

### 3.3. Cytotoxic activity

The cytotoxic activity was determined by MTT assay according to the protocol previously described.^30^ The cells were cultured in RPMI-1640 (Serana MCL-041, Germany), supplemented with 20% fetal bovine serum and 1% penicillin/streptomycin. They were seeded in a 96 well plate at 1 × 10^5^ cells per mL (100 μL per well) and incubated at 37 °C for 24 h in 5% CO_2_ atmosphere and 100% humidity. After cell confluency, the growth medium was removed and the cell monolayer was washed twice with media. NC was solubilized in DMSO at 10 mM concentration, 10 μL of the former stock solution was diluted with 90 μL RPMI medium containing 2% serum (maintenance medium). Half-fold serial dilutions of NC were prepared in the same medium, of which 10 μL were transferred to each well to make up 100 μL of the culture. Each concentration (final concentrations are 100, 50, 25, 12.5 and 6.25 μM) was investigated in 3 different wells leaving 3 wells as control, which received only the maintenance medium. The cells were incubated at 37 °C for 24 h, then 20 μL of MTT solution (5 mg mL^−1^, Bio Basic Canada Inc.) were added to each well, gently mixed by shaking at 150 rpm for 5 min. The cells were further incubated at 37 °C for 4 h. Media was removed and 200 μL DMSO were added. The plate was shaken at 150 rpm for 5 min to thoroughly solubilize the formazan (MTT metabolic product). The optical density was determined at 560 nm, where it is directly correlated with viable cell quantity.

For quantification of EGFR and TNF, the cells were incubated with NC at IC_50_ for 24 h under the above condition. Cell culture supernatants were then collected and quantitative detection of EGFR and TNF-α was performed by Bioassay Technology Laboratory Human Epidermal Growth Factor Receptor Sandwich Kit (Cat. No. E0313Hu) and Bioassay Technology Laboratory Human Tumor Necrosis Factor Sandwich Kit (Cat. No. E0082Hu), respectively, according to the manufacturer's instructions, the detailed procedures are available in the ESI.†

### 3.4. Antiviral activity

The antiviral activity was determined by MTT assay according to the protocol previously described.^31^ Vero cell line ATCC CCL-81 was obtained from VACSERA, Agouza, Egypt. Herpes simplex-I (HSV-I) virus was obtained from the Department of Microbiology, Faculty of Medicine, Al-Azhar University (Girls), Egypt. Dulbecco's Modified Eagle Medium (DMEM) was supplemented with 10% FBS, 100 units per mL of penicillin, 100 mg mL^−1^ of streptomycin, 0.07% NaHCO_3_ and 2 mM l-glutamine.

Vero cells (1 × 10^5^ cells per mL), seeded in DMEM media, were incubated in a 96 well plate at 37 °C until confluency. The CC_50_ and MNTC for NC were determined using MTT following the same conditions described for cytotoxicity assay.

For assessment of the antiviral activity, Vero cells (1 × 10^4^ cells per mL), seeded in DMEM (200 μL), were incubated at 37 °C in 5% CO_2_ till confluency. Serial dilutions of the MNTC of NC were prepared. Equal volume (1 : 1 v/v) of NC and viral suspension were incubated for 1 h. The NC/viral suspension (100 μL) was added to Vero cells and incubated at 37 °C in 5% CO_2_ atmosphere for 24 h. Cell viability was determined by MTT as described above.

### 3.5. Statistical analysis

All results were expressed as mean ± standard deviation of the mean (mean ± SD). Normality was checked using the Shapiro–Wilk test. Student's *t*-test was used to analyze statistically significant differences between the two groups. The statistical analysis was conducted using GraphPad Prism software (v. 8.0.2).

### 3.6. In *silico* protocols

The structure of NC was drawn using ChemDraw software (v. 22.0.0) and saved as a 3D structure in MOL format. Maestro software was used for the docking procedure, and the crystal structures of proteins were retrieved from the Protein Data Bank in PDB format (PDB: 2A4L^32^ and 2KI5 (ref. 33)), both proteins were prepared, and the Glide program was used to dock NC into their active sites. The best docking pose was used as input for molecular dynamic simulations using Desmond software (Schrodinger CC; details are given in the ESI†). The DFT calculations were performed using Gaussian 16 software,^34^ and the MOL file was used as input for these calculations. Frequency calculations followed the geometry calculations to ensure that the structure was at a minimum with no negative frequency. All calculations were at B3LYP/6-31+G*/Gas level,^35^ GaussView^34,36^ and Pymol^36^ software were used for *in silico* visualization.

## 4. Conclusion

In summary, a new dimeric sesquineolignan, designated nuciferol C, was isolated from the endocarp of *Cocos nucifera* L. Nucifrol C exerted potential cytotoxic effect against colon cancer, and significantly decreased EGFR and TNF-α, indicating potential anticancer properties that should be further assessed. In addition, nuciferol C exhibited antiviral effect against herpes simplex type-1. Molecular docking studies against both herpes simplex type-1 thymidine kinase and cyclin-dependent kinase 2 suggest that the antiviral activity of nuciferol C is *via* cyclin-dependent kinase 2 inhibition. Molecular dynamic simulations showed that nuciferol C is stable inside the active site and was able to hold hydrogen bonding within it. Finally, DFT calculation was implemented to study the structure and its electronic properties. The findings suggest that nuciferol C is an additional lignan candidate for anticancer and antiviral agents. It also supports that underexplored coconut parts are fruitful reservoir for discovering new metabolites.

## Data availability

Data supporting this study are openly available from Marwa Elsbaey

## Author contributions

Marwa Elsbaey: conceptualization, investigation, methodology, writing – review & editing; Radwan Alnajjar and Khaled M. Darwish: investigation, formal analysis, software, writing – review & editing; Yasuhiro Igarashi and Tomofumi Miyamoto: supervision.

## Conflicts of interest

There are no conflicts to declare.

## Supplementary Material

RA-014-D4RA02940B-s001
