# Supplementary material for: Nuciferol C, a new sesquineolignan dimer from Cocos nucifera L.: bioactivity and theoretical investigation
Source: RSC Adv. 2024 Aug 16;14(36):25900–7. doi: 10.1039/d4ra02940b (PMC11328675; doi:10.1039/d4ra02940b)
Supplement: RA-014-D4RA02940B-s001 [file RA-014-D4RA02940B-s001.pdf]

Supplementary Information (SI) for RSC Advances.
This journal is © The Royal Society of Chemistry 2024

Supporting Information for

Nuciferol C, a new sesquineolignan dimer from *Cocos nucifera* L.: bioactivity and theoretical investigation

Marwa Elsbaey[a*], Yasuhiro Igarashi[b], Radwan Alnajjar[c], Khaled M. Darwish[d], Tomofumi Miyamoto[e]

[a] Department of Pharmacognosy, Faculty of Pharmacy, Mansoura University, Mansoura 35516, Egypt.

[b] Biotechnology Research Center and Department of Biotechnology, Toyama Prefectural University, 5180 Kurokawa, Imizu, Toyama, 939-0398, Japan.

[c] CADD Unit, Faculty of Pharmacy, Libyan International Medical University, Benghazi, 16063, Libya.

[d] Department of Medicinal Chemistry, Faculty of Pharmacy, Suez Canal University, Ismailia 41522, Egypt Graduate.

[e] School of Pharmaceutical Sciences, Kyushu University, 3-1-1 Maidashi, Higashi-ku, Fukuoka 812-8582, Japan.

*Corresponding author: Marwa Elsbaey; marwaelsebay1611@mans.edu.eg

Table of contents

Content	Page
**Figure S1**: ^{1}H-NMR spectrum of nuciferol C (600 MHz, CD_3OD).	1
**Figure S2**: ^{13}C-NMR spectrum of nuciferol C (150 MHz, CD_3OD).	2
**Figure S3**: HSQC spectrum of nuciferol C (600 MHz, CD_3OD).	3
**Figure S4**: HMBC spectrum of nuciferol C (600 MHz, CD_3OD).	4
**Figure S5**: COSY spectrum of nuciferol C (600 MHz, CD_3OD).	5
**Figure S6**: TCOSY spectrum of nuciferol C (600 MHz, CD_3OD).	6
**Figure S7**: NOESY spectrum of nuciferol C (600 MHz, CD_3OD).	7
**Figure S8**: Mass spectrum of nuciferol C (600 MHz, CD_3OD).	8
**Figure S9**: Negative HRESITOF mass spectrum of nuciferol C (600 MHz, CD_3OD).	8
**Figure S10**: UV spectrum of nuciferol C.	9
**Figure S11**: IR spectrum of nuciferol C.	10
**Figure S12**: ^{1}H-NMR spectrum of nuciferol A (600 MHz, CD_3OD).	11
**Figure S13**: ^{1}H-NMR spectrum of nuciferol B (600 MHz, CD_3OD).	12
**Table S1**: NMR data of nuciferol C as compared to nuciferols A and B.	13
**Figure S14**: Proposed dimerization of nuciferol B into nuciferol C through oxidative coupling, adapted from Sotheeswaran & Pasupathy's proposed biosynthesis of the oligmeric stilbene hopeaphenol[1].	14
Experimental	15
**Table S2**. Antiviral and cytotoxic activities of nuciferol C.	17

Figure S1: ^{1}H-NMR spectrum of nuciferol C (600 MHz, CD_3OD).

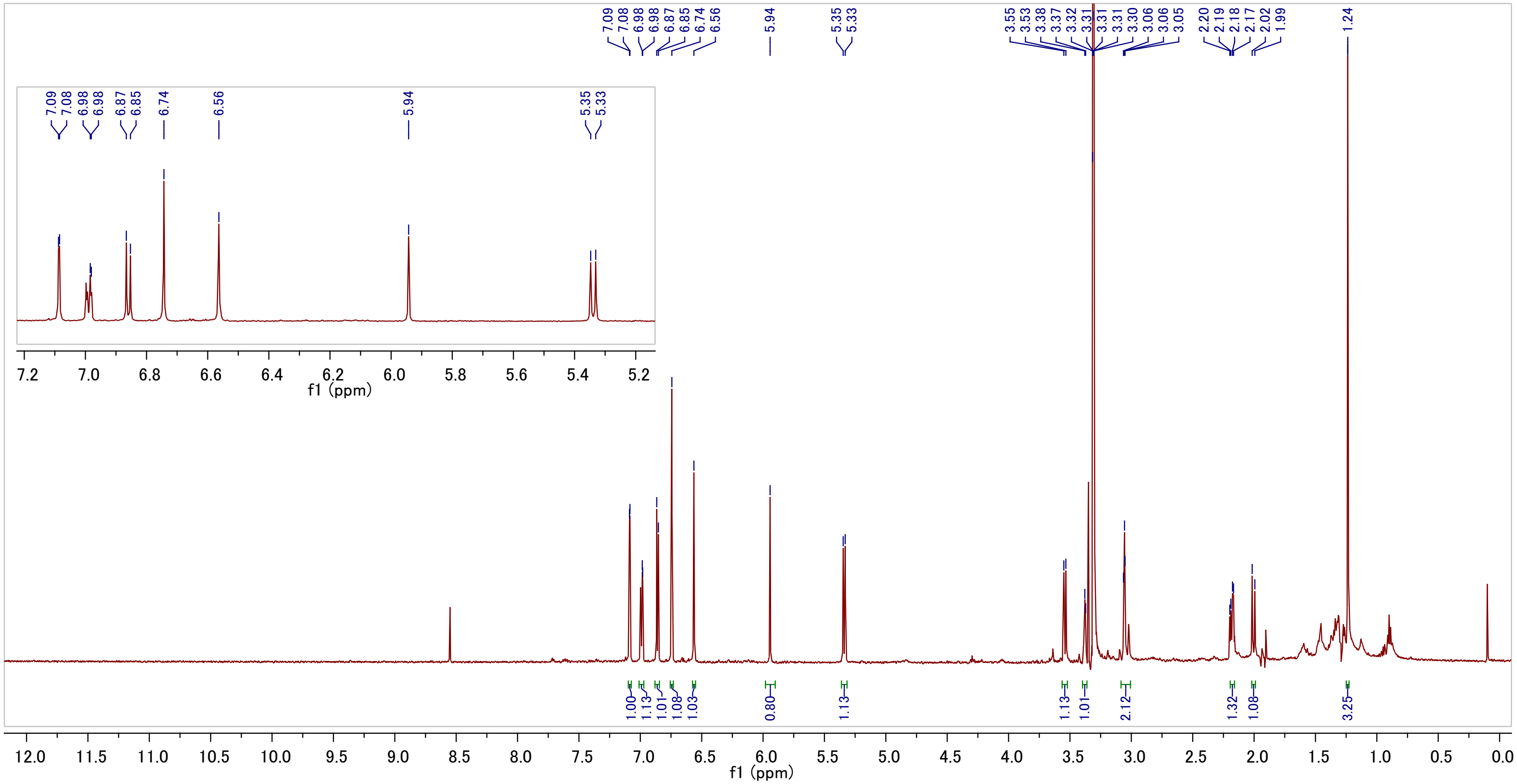

Figure S2: ^{13}C-NMR spectrum of nuciferol C (150 MHz, CD_3OD).

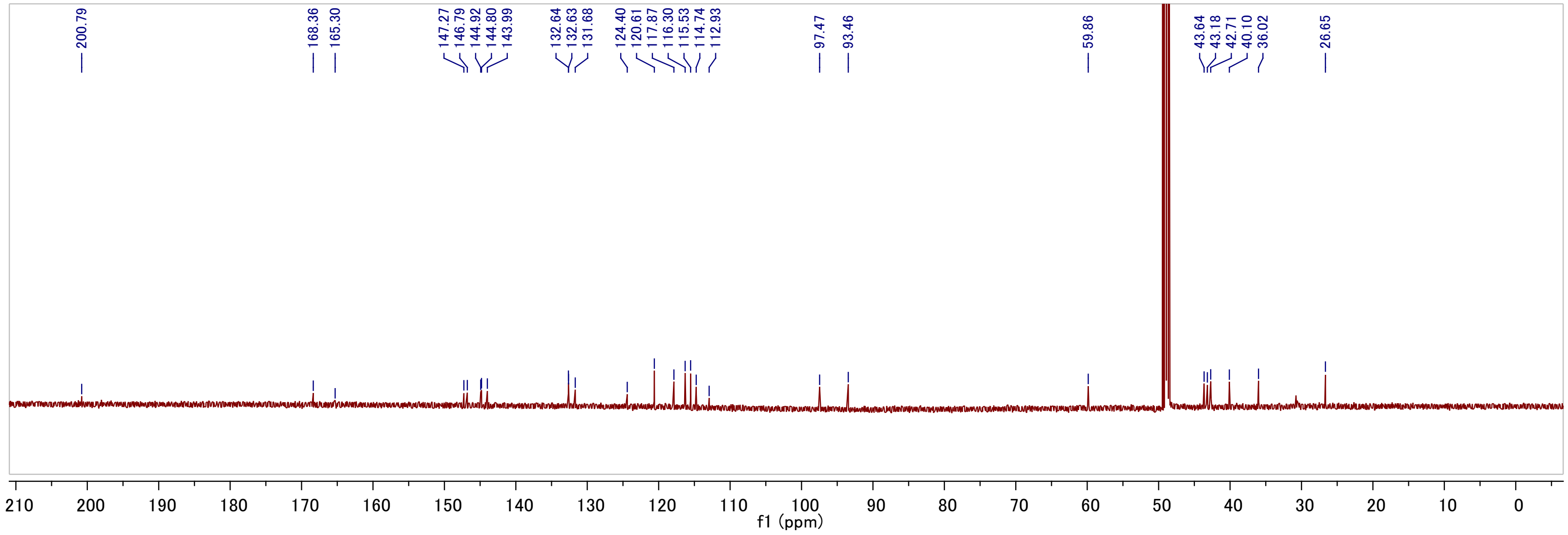

Figure S3: HSQC spectrum of nuciferol C (600 MHz, CD_3OD).

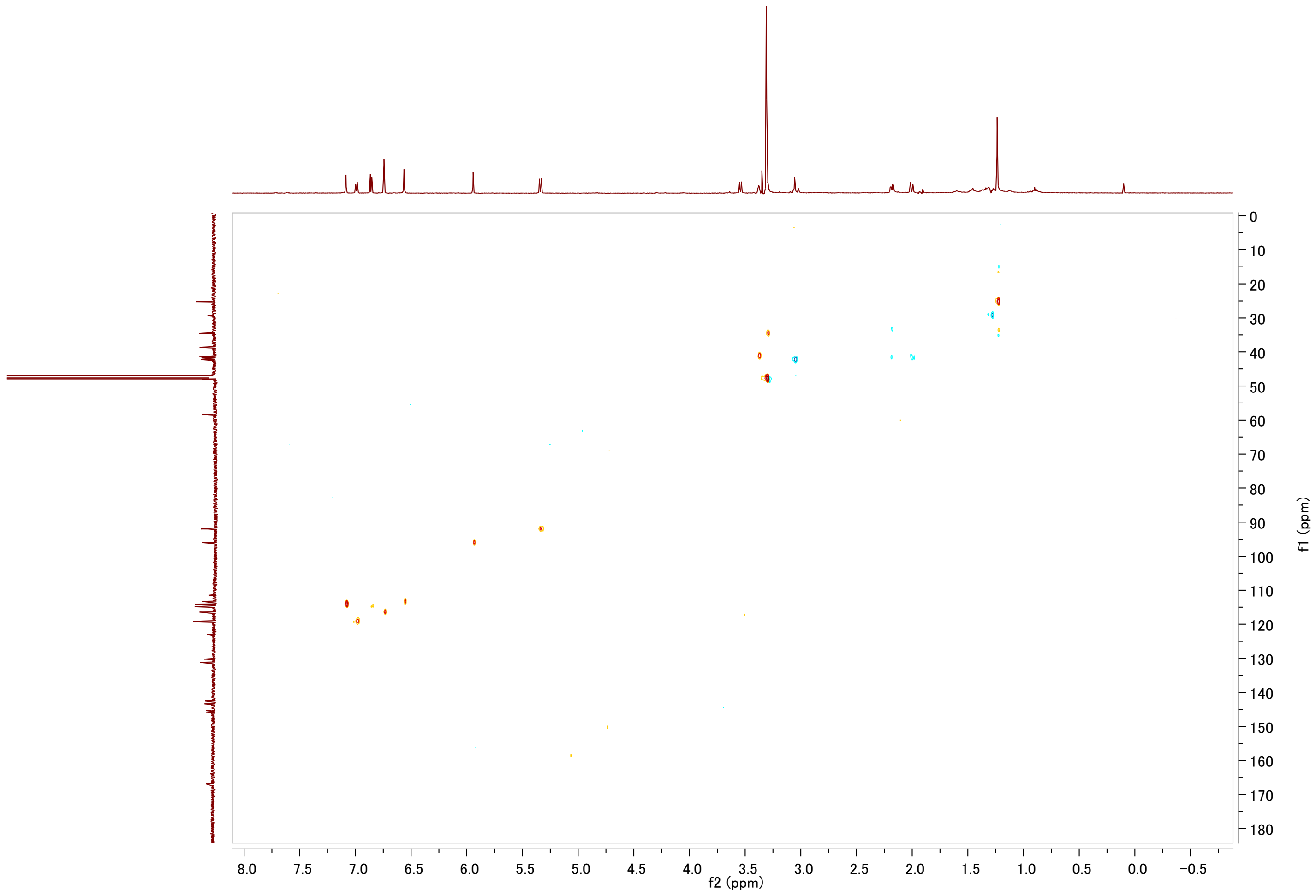

Figure S4: HMBC spectrum of nuciferol C (600 MHz, CD_3OD).

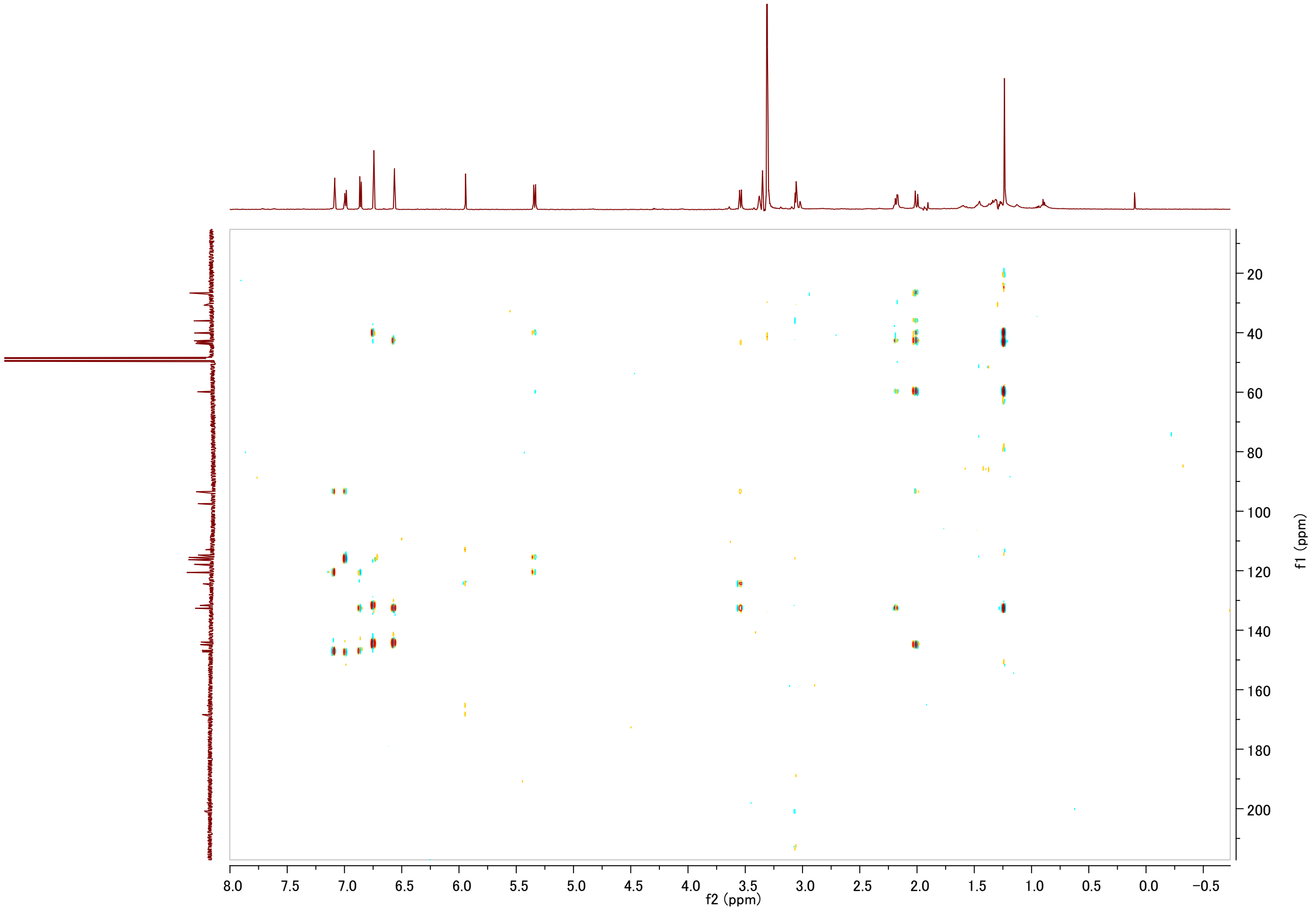

Figure S5: COSY spectrum of nuciferol C (600 MHz, CD_3OD).

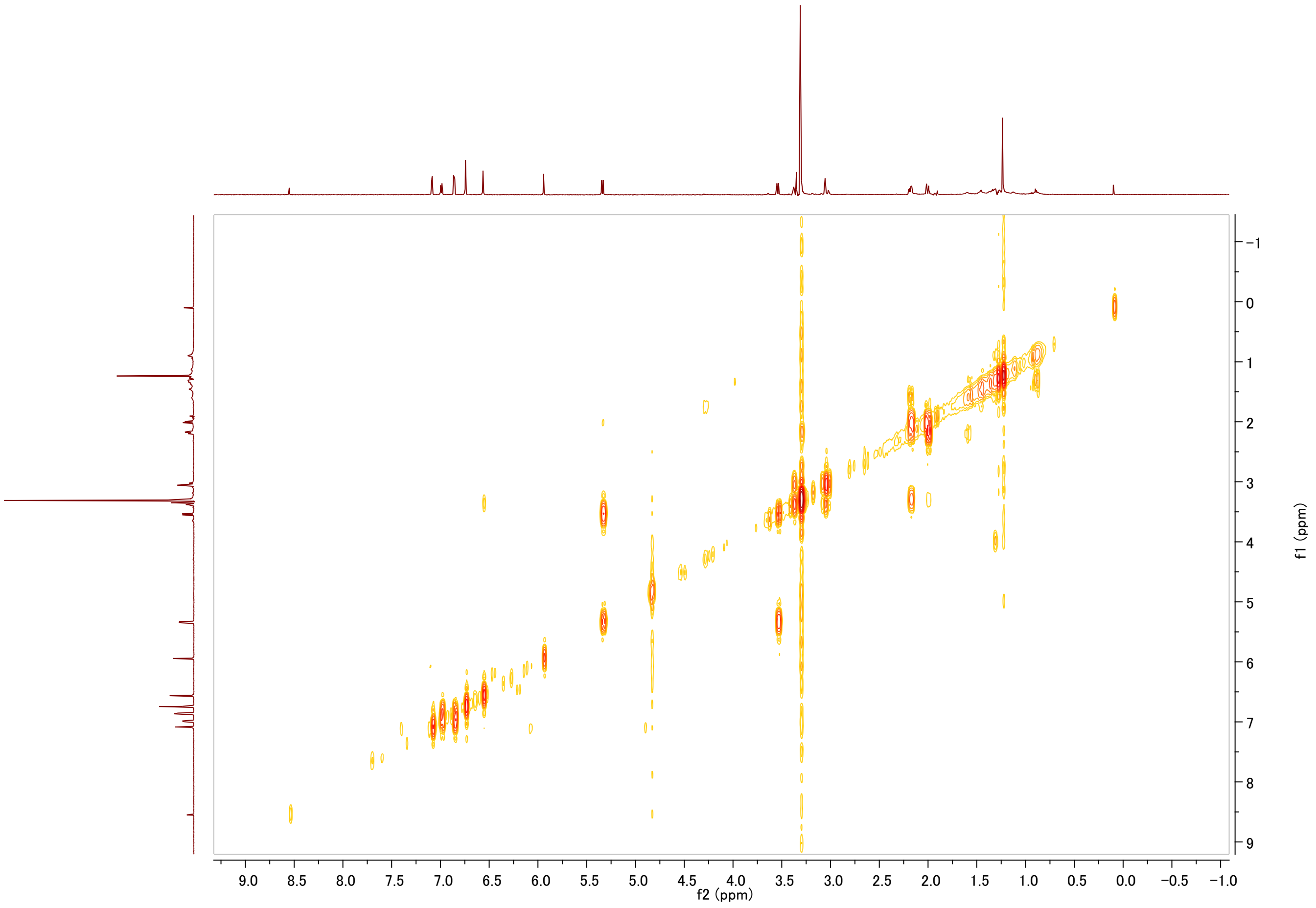

Figure S6: TCOSY spectrum of nuciferol C (600 MHz, CD_3OD).

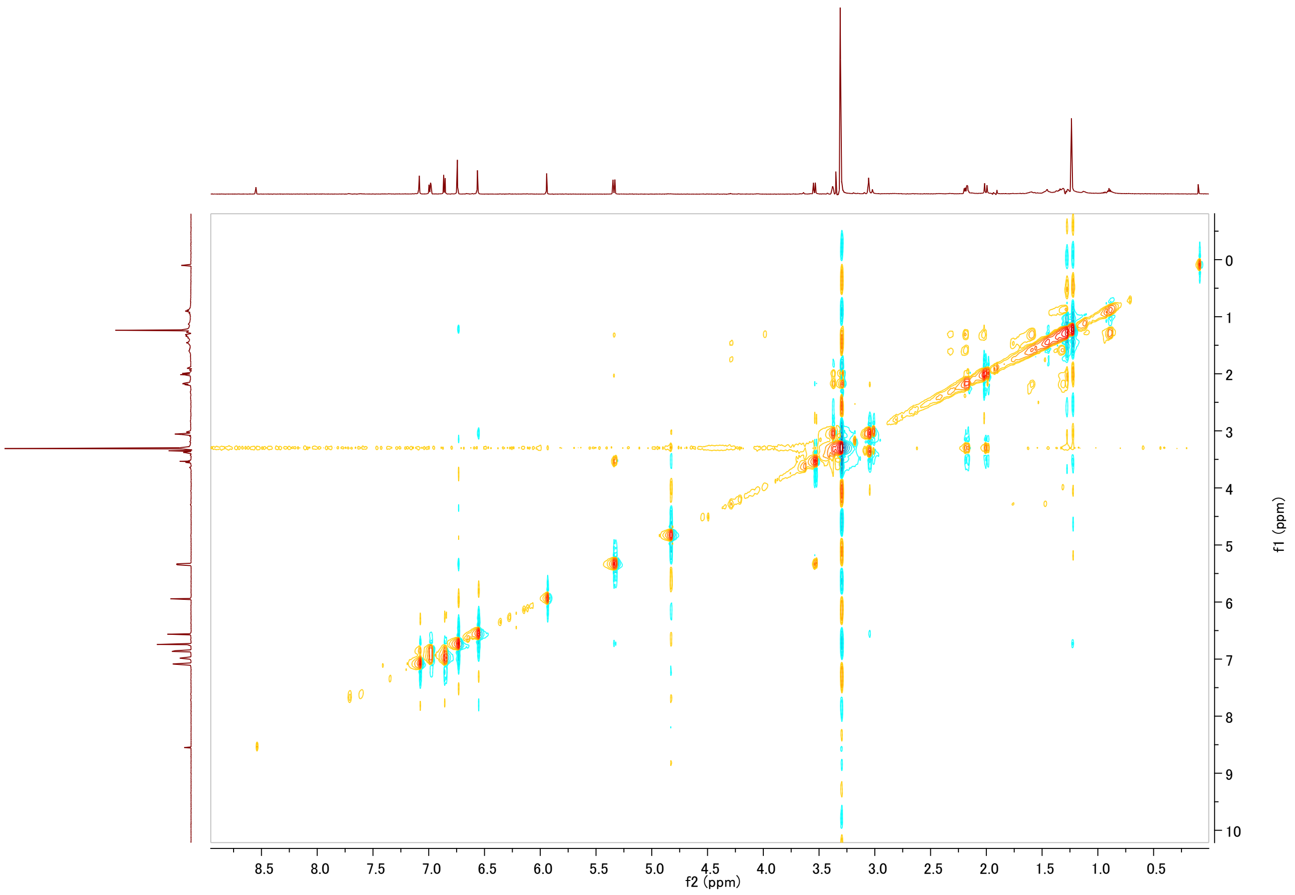

Figure S7: NOESY spectrum of nuciferol C (600 MHz, CD_3OD).

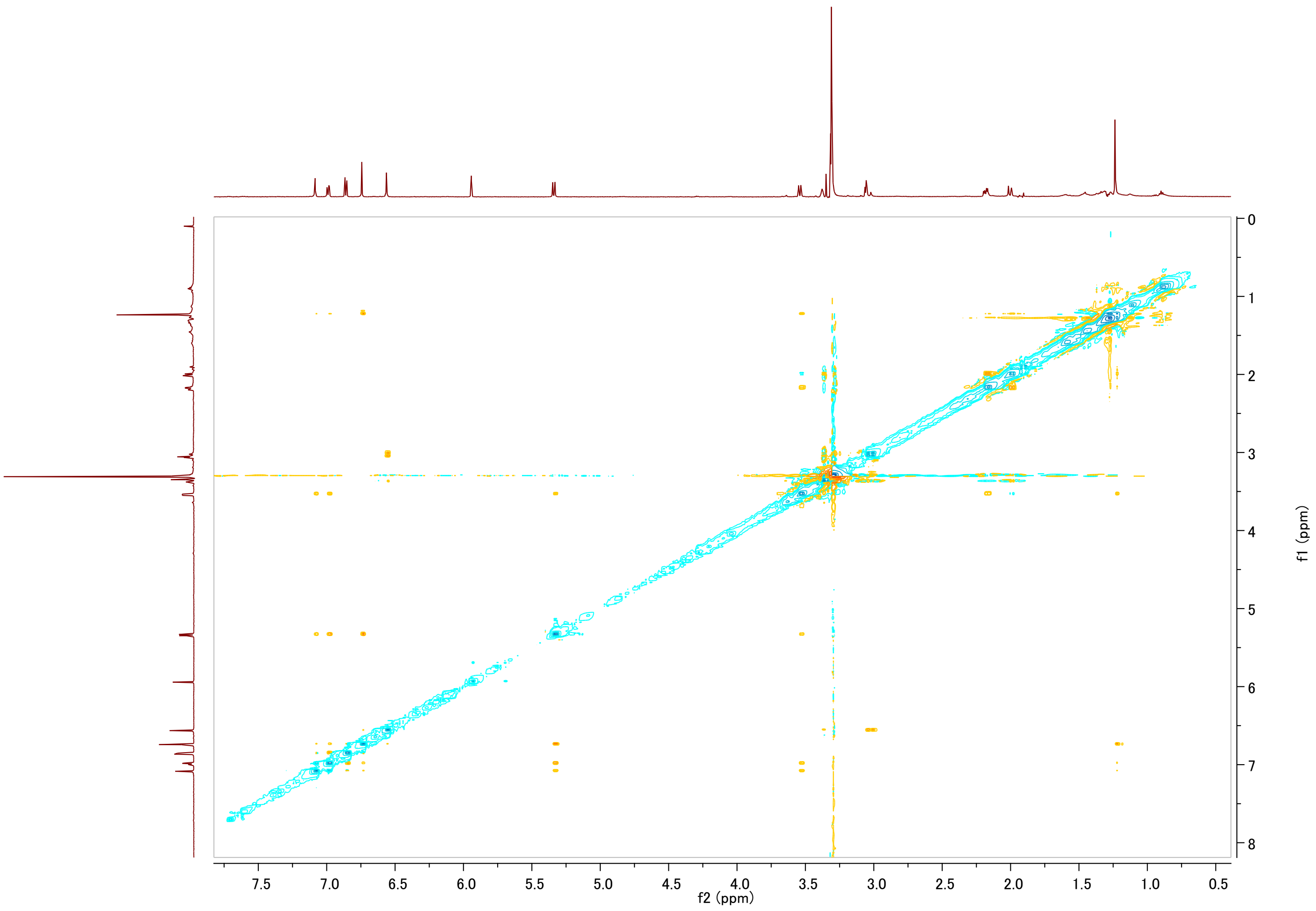

Figure S8: Mass spectrum of nuciferol C.

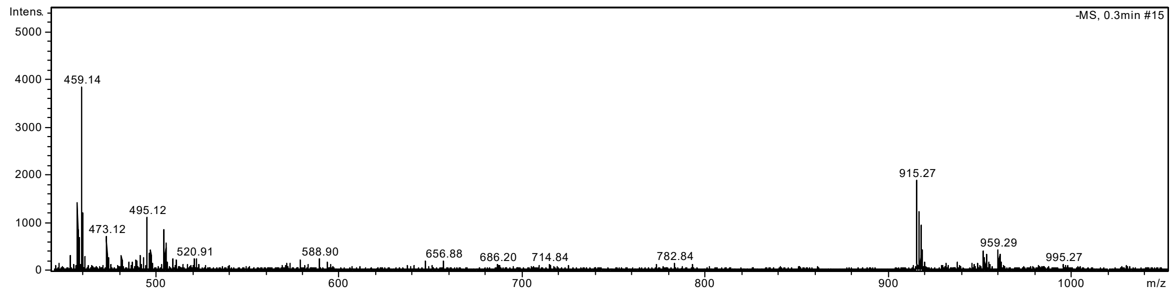

Figure S9: Negative HRESITOF mass spectrum of nuciferol C.

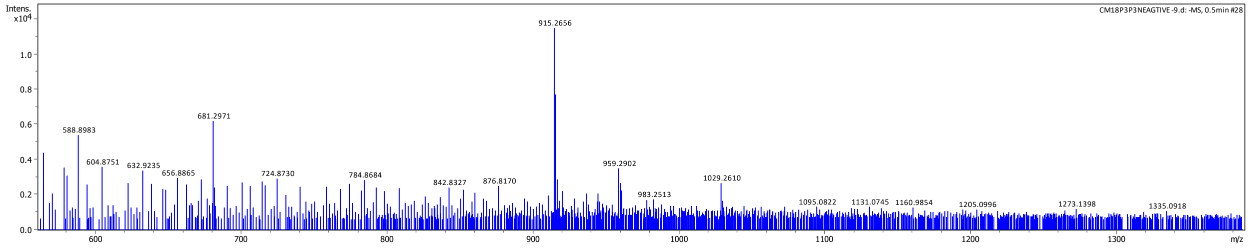

Figure S10: UV spectrum of nuciferol C in methanol.

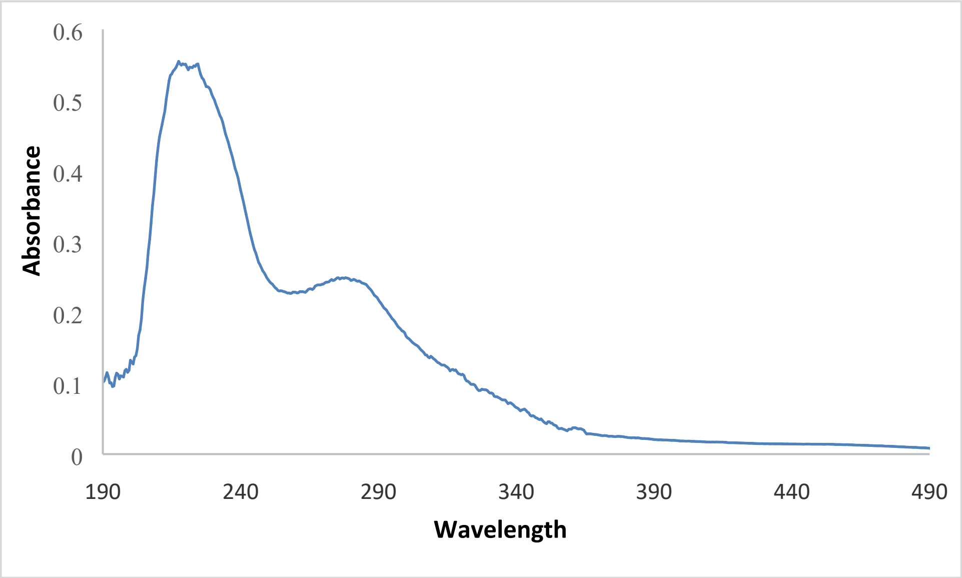

Figure S11: IR spectrum of nuciferol C.

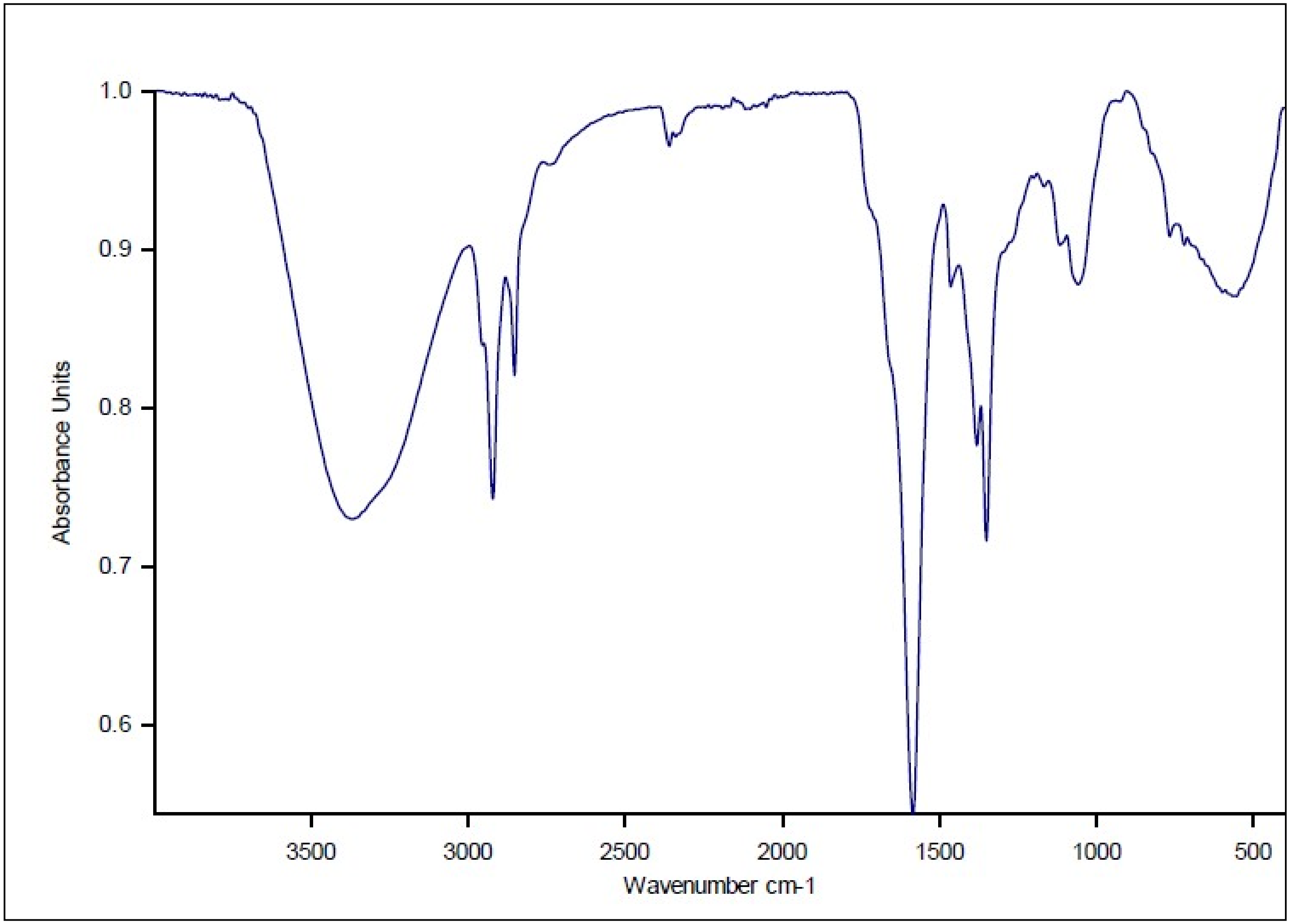

Figure S12: ^{1}H-NMR spectrum of nuciferol A (600 MHz, CD_3OD).

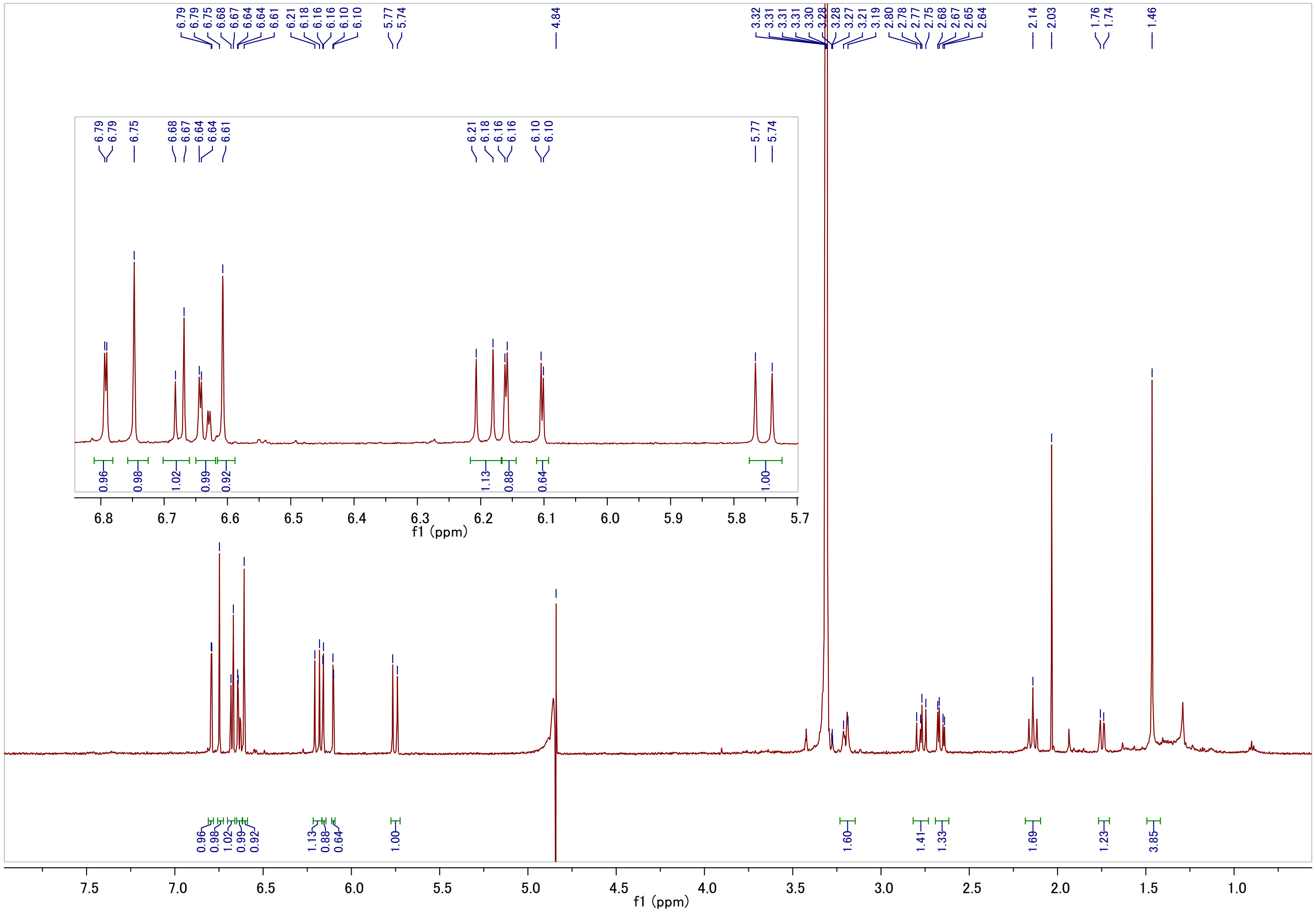

Figure S13: ^{1}H-NMR spectrum of nuciferol B (600 MHz, CD_3OD).

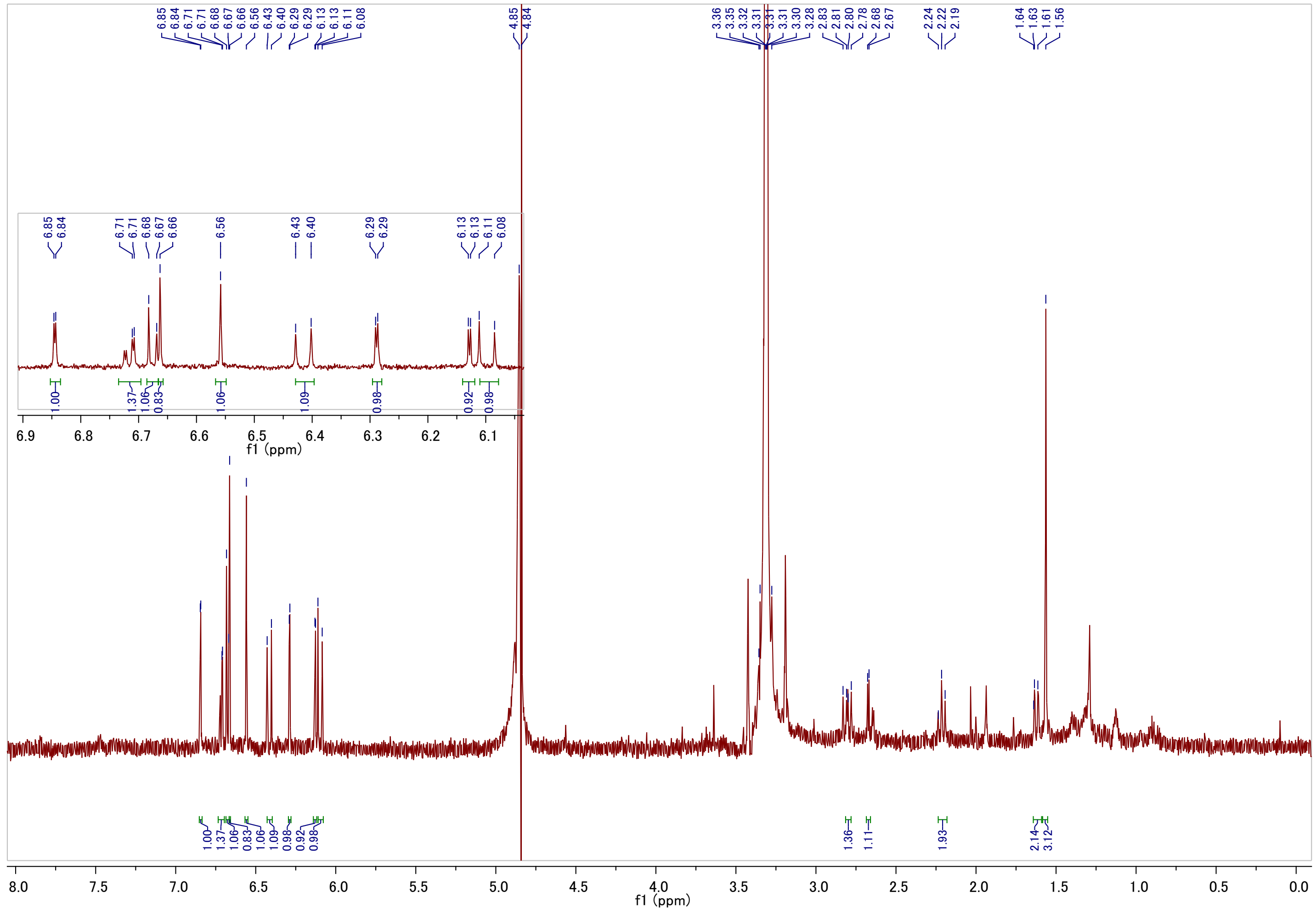

Table S1: NMR data of nuciferol C as compared to nuciferols A and B[2].

	**Nuciferol A**		**Nuciferol B**		**Nuciferol C**	
#	$^{\delta}$C, mult.	δ_H, mult (*J* in Hz)	^{13}C-NMR	δ_H, mult (*J* in Hz)	$^{\delta}$C, mult.	δ_H, mult (*J* in Hz)
1	109.4, C	-----	108.6, C	-----	112.9, C	-----
2	165.8, C	-----	165.5, C	-----	168.4, C	-----
3	100.3, CH	6.09, d (2.4)	100.8, CH	6.12, d (2.4)	97.5, CH	5.94, s
4	165.2, C	-----	168.8, C	-----	165.3, C	-----
5	107.3, CH	6.14, d (2.4)	108.5, CH	6.28, d (2.4)	**124.4, C**	-----
6	152.1, C	-----	151.7, C	-----	144.8, C	-----
7	201.7, C	-----	200.8, C	-----	200.8, C	-----
8 α	42.6, CH_2	2.64, dd (5.4, 18.0)	42.6, CH_2	2.65, dd (4.8, 17.8)	43.6, CH_2	3.06, m
8 β		2.75, dd (13.4, 18.0)		2.79, dd (13.8, 18.0)		-----
9	37.2, CH	3.28*	37.2, CH	3.34 *	42.7, CH	3.38, m
1’	131.4, C	-----	133.1, C	-----	132.63, C	-----
2’	114.6, CH	6.74, s	115.1, CH	6.65, s	117.9, CH	6.74, s
3’	143.7, C	-----	143.6, C	-----	144.0, C	-----
4’	143.8, C	-----	143.8, C	-----	144.9, C	-----
5’	114.3, CH	6.60, s	114.4, CH	6.55, s	114.7, CH	6.56, s
6’	130.4, C	-----	129.4, C	-----	131.7, C	-----
7’	40.9, C	-----	40.7, C	-----	40.1, C	-----
8’ α	41.2, CH_2	1.73, dd (1.8, 13.2)	40.9, CH_2	1.61, br.d (13.2)	43.2, CH_2	2.00, d (13.2)
8’ β		2.12, t (13.2)		2.20, t (13.8)		2.18, dd (5.4, 13.2)
9’	35.3, CH	3.18, dt (2.4, 13.2)	35.4, CH	3.36 *	36.0, CH	3.31*
1’’	129.7, C	-----	129.9, C	-----	132.64, C	-----
2’’	112.2, CH	6.78, d (1.8)	112.2, CH	6.84, d (1.8)	115.5, CH	7.08, d (2)
3’’	144.8, C	-----	144.8, C	-----	146.8, C	-----
4’’	144.4, C	-----	144.4, C	-----	147.3, C	-----
5’’	114.8, CH	6.66, d (8.4)	114.8, CH	6.68, d (8.4)	116.3, CH	6.86, d (8.1)
6’’	118.0, CH	6.63, dd (2.4, 8.4)	118.1, CH	6.71, br. dd (1.8, 8.4)	120.6, CH	6.98, dd (2.1, 8.1)
7’’	129.1, CH	5.74, d (15.6)	126.3, CH	6.4, d (16.2)	**93.5, CH**	**5.34, d (9.9)**
8’’	137.7, CH	6.18, d (15.6)	137.1	6.10, d (15.6)	**59.9, CH**	**3.54, d (9.9)**
9’’	28.6	1.45, s	27.6	1.55, s	26.7, CH_3	1.24, s

*masked by solvent signal, determined through HSQC

Figure S14: Proposed dimerization of nuciferol B into nuciferol C through oxidative coupling, adapted from Sotheeswaran & Pasupathy's proposed biosynthesis of the oligmeric stilbene hopeaphenol[1].

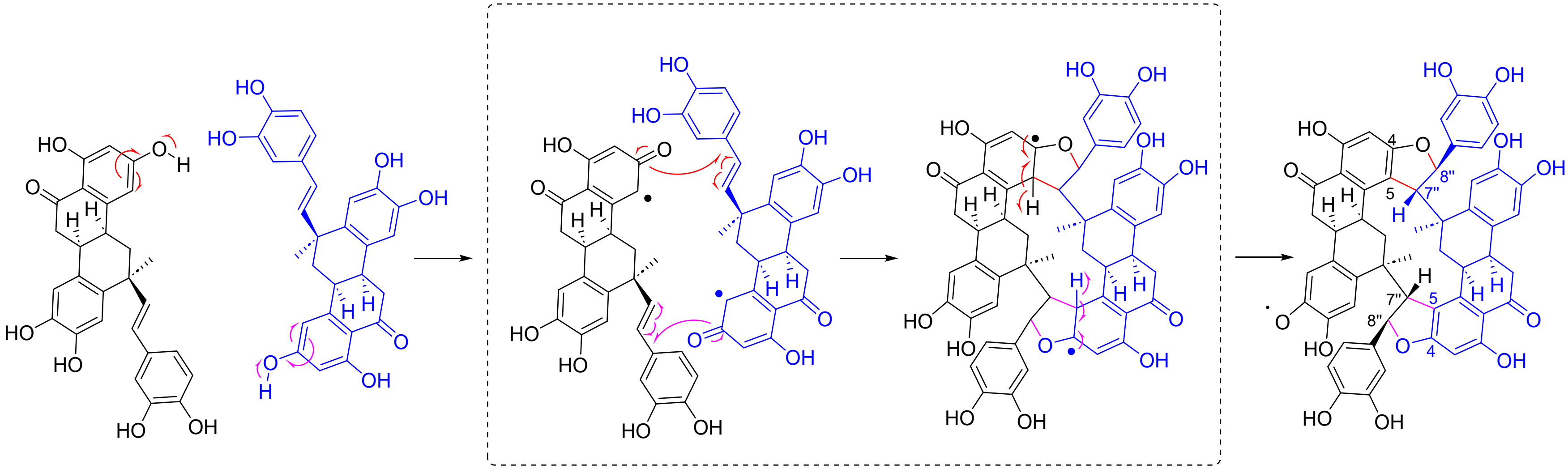

Nuiferol B

phenoxy radical intermediates

Nuiferol C

Experimental

Bioactivity

Quantitative detection of EGFR and TNF-α was performed by the Bioassay Technology Laboratory Human Epidermal Growth Factor Receptor Sandwich Kit (Cat. No. E0313Hu) according to manufacturer's instructions.

Principle

This kit is an Enzyme-Linked Immunosorbent Assay (ELISA). The micro ELISA plate provided in this kit is pre-coated with Human EGFR antibody. Standard or sample containing EGFR is added where it binds to antibodies coated on the wells. Then biotinylated Human EGFR Antibody is added, accordinly it binds with EGFR. Then Streptavidin- Horseradish Peroxidase (HRP) is added to bind to the Biotinylated EGFR antibody. Free components are washed away. The substrate solution is added to each well. Only those wells that contain Human EGFR, biotinylated detection antibody and Streptavidin-HRP conjugate will appear blue in color. The enzyme-substrate reaction is terminated by the addition of stop solution and the color turns yellow. The optical density (OD) is measured spectrophotometrically at a wavelength of 450 nm ± 2 nm. The OD value is proportional to the concentration of Human EGFR. The concentration of Human EGFR in the sample can be calculated by comparing the OD of the samples to the standard curve.

Procedures:

All reagents are brought to room temperature before use. The assay is performed at room temperature. The standard solutions (50 μL) are added to standard wells. The cell supernatents (40 μL), folowed by anti-EGFR antibody (10 μL) are added to the sample wells. Streptavidin-HRP (50 μL) is added to both standard and sample wells but not to the blank cells. After gentle shanking, the plate is covered with a sealer and incubated for 60 min at 37 ^{0} C. The wells are washed 5 times with wash buffer i.e. the wash buffer (300 μL) was added for 30 sec to 1 min for each wash. The plate is ploted onto a paper towel to remove any remaing washing buffer, the subatrate solution A (50 μL) is added to each well

followed by the subatrate solution B (50 μL). the plate is covered with a sealer and incubated for 10 min at 37 ^{0} C in the dark to allow blue color developpment. The stop solution (50 μL) is added to each well, the blue color will be changed to yellow. The OD value of each well is immediately determined using a microplate reader set to 450 nm within 10 min. after adding the stop solution. The standard curve is constructed by plotting the average OD for each standard on the vertical (Y) axis against the concentration on the horizontal (X) axis.

Quantitative detection of TNF-α was performed by the Bioassay Technology Laboratory Human Tumor necrosis factor Sandwich Kit (Cat. No. E0082Hu) according to manufacturer's instructions.

This kit is employs an ELISA-Sandwitch priniciple. A micro ELISA plate has been pre-coated with Human TNF-α antibody. TNF-α present in the sample/standard is added to bind to antibodies coated on the wells. And then biotinylated Human TNF-α Antibody is added where it binds to TNF-α in the sample. Then Streptavidin-HRP is further added to bind to the Biotinylated TNF-α antibody. After incubation unbound Streptavidin-HRP is washed away during a washing step. Substrate solution is then added and color develops in proportion to the amount of Human TNF-α. The reaction is terminated by addition of acidic stop solution and absorbance is measured at 450 nm.

Procedures:

This was done according to the same procedures decribed above for EGFR.

Table S2. Antiviral and cytotoxic activities of nuciferol C (NC).

	Antiviral activity				Cytotoxicity in Caco-2		
	Vero cells		HSV-I		Cytotoxicity	TNF-α	EGFR
	CC_{50} (µM)	MNTC (µM)	IC_{50} (µM)	SI (CC_{50}/IC_{50})	IC_{50} (µM)	Conc (ng/ml)	Conc (ng/ml)
**NC**	75.91 ± 0.08	31.25	23.4 ± 0.74	3.2	26.55 ± 0.66	78.8 ± 0.57	14.52 ± 0.17
**Control**	-	-	-	-	-	52.5 ± 3.2	23.88 ± 0.09

CC_{50}: 50 % cytotoxic concentration; MNTC: maximum nontoxic concentration; IC_{50}: 50 % inhibition concentration; SI: selectivity index

Docking Study

The protein was retrieved from the Protein Data Bank in PDB format Human cyclin-dependent kinase 2 (PDB: 2A4L2)[3] and herpes simplex type-1 thymidine kinase (2KI5),[4] Next, both proteins were prepared using the protein preparation panel using the default setting. At first, nuciferol C were docked into the active site of each protein using standard precision (SP) glide mode using the default setting with the OPLS4 force field (Schrodinger 2021-3).

Molecular Dynamics simulations

The MD simulations were carried out using Desmond simulation package of Schrödinger LLC.[5] The NPT ensemble with the temperature 300 K and a pressure 1 bar was applied in all runs. The simulation length was 500 ns with a relaxation time 1 ps. The OPLS4 force field parameters were used in all simulations.[6] The cutoff radius in Coulomb interactions was 20.00Å. The orthorhombic periodic box boundaries were set 10 Å away from the protein atoms., The water molecules were explicitly described using the transferable intermolecular potential with three points (TIP3P) model.[7] salt concentration set to 0.15 M NaCl and was built using the System Builder utility of Desmond.[5] The Martyna−Tuckerman−Klein chain coupling scheme with a coupling constant of 2.0 ps was

used for the pressure control and the Nosé−Hoover chain coupling scheme for the temperature control.[8] Nonbonded forces were calculated using a RESPA integrator where the shortrange forces were updated every step and the long-range forces were updated every three steps. The trajectories were saved at 20 ns intervals for analysis. The behavior and interactions between the ligands and protein were analyzed using the Simulation Interaction Diagram tool implemented in Desmond MD package. The stability of MD simulations was monitored by looking on the RMSD of the ligand and protein atom positions in time.

References

1. S. S Velu, N. F Thomas and J.-F. F Weber, *Current Organic Chemistry*, 2012, **16**, 605-662.
2. M. Elsbaey, B. Jie, C. Tanaka, H. Kato, S. Tsukamoto, K. Usui, G. Hirai and T. Miyamoto, *Tetrahedron letters*, 2019, **60**, 150948.
3. W. F. De Azevedo, S. Leclerc, L. Meijer, L. Havlicek, M. Strnad and S.-H. Kim, 1997, **243**, 518-526.
4. M. S. Bennett, F. Wien, J. N. Champness, T. Batuwangala, T. Rutherford, W. C. Summers, H. Sun, G. Wright and M. R. Sanderson, 1999, **443**, 121-125.
5. S. J. D. S. R. Release, New York, NY, 2017.
6. C. Lu, C. Wu, D. Ghoreishi, W. Chen, L. Wang, W. Damm, G. A. Ross, M. K. Dahlgren, E. Russell, C. D. J. J. o. c. t. Von Bargen and computation, 2021, **17**, 4291-4300.
7. W. L. Jorgensen, J. Chandrasekhar, J. D. Madura, R. W. Impey and M. L. J. T. J. o. c. p. Klein, 1983, **79**, 926-935.
8. G. J. Martyna, M. L. Klein and M. J. T. J. o. c. p. Tuckerman, 1992, **97**, 2635-2643.
